# Interleukin-27 controls basal pain threshold in physiological and pathological conditions

**DOI:** 10.1038/s41598-018-29398-3

**Published:** 2018-07-23

**Authors:** Tomoko Sasaguri, Toru Taguchi, Yuzo Murata, Kimiko Kobayashi, Sayaka Iizasa, Ei’ichi Iizasa, Makoto Tsuda, Naomi Hirakawa, Hiromitsu Hara, Hiroki Yoshida, Toshiharu Yasaka

**Affiliations:** 1 Department of Anesthesiology & Critical Care Medicine, Faculty of Medicine, Saga University, 5-1-1 Nabeshima, Saga, 849-8501 Japan; 2 Department of Physical Therapy, Niigata University of Health and Welfare, 1398 Shimami-cho, Kita-ku, Niigata, 950-3198 Japan; 3 Department of Neuroscience II, Research Institute of Environmental Medicine, Nagoya University, Furo-cho, Chikusa-ku, Nagoya 464-8601 Japan; 4 Division of Histology and Neuroanatomy, Department of Anatomy & Physiology, Faculty of Medicine, Saga University, 5-1-1 Nabeshima, Saga, 849-8501 Japan; 5 Department of Anatomy and Neuroscience, Hyogo College of Medicine, 1-1 Mukogawa-cho, Nishinomiya, Hyogo, 663-8501 Japan; 6 Department of Biological Science and Technology, The United Graduate School of Agricultural Sciences, Kagoshima University, 1-21-24 Korimoto, Kagoshima, 890-8580 Japan; 7 Department of Immunology, Graduate School of Medical and Dental Sciences, Kagoshima University, 8-35-1 Sakuragaoka, Kagoshima, 890-8544 Japan; 8 Department of Molecular and System Pharmacology, Graduate School of Pharmaceutical Science, Kyushu University, 3-1-1 Maidashi, Higashi-ku, Fukuoka, 812-8582 Japan; 9 Division of Molecular and Cellular Immunoscience, Department of Biomolecular Sciences, Faculty of Medicine, Saga University, 5-1-1 Nabeshima, Saga, 849-8501 Japan

## Abstract

Numerous studies have shown that pain sensation is affected by various immune molecules, such as cytokines, in tissues comprising the sensory pathway. Specifically, it has been shown that interleukin (IL)-17 promotes pain behaviour, but IL-10 suppresses it. IL-27 has been reported to have an anti-inflammatory effect through regulation of T cell differentiation, resulting in reduced IL-17 and induction of IL-10. Thus, we hypothesised that IL-27 would have some regulatory role in pain sensation. Here, we provide evidence that endogenous IL-27 constitutively controls thresholds for thermal and mechanical sensation in physiological and pathological conditions. Mice lacking IL-27 or its receptor WSX-1 spontaneously showed chronic pain-like hypersensitivity. Reconstitution of IL-27 in IL-27-deficient mice reversed thermal and mechanical hypersensitive behaviours. Thus, unlike many other cytokines induced by inflammatory events, IL-27 appears to be constitutively produced and to control pain sensation. Furthermore, mice lacking IL-27/WSX-1 signalling showed additional hypersensitivity when subjected to inflammatory or neuropathic pain models. Our results suggest that the mechanisms underlying hypersensitive behaviours caused by the ablation of IL-27/WSX-1 signalling are different from those underlying established chronic pain models. This novel pain control mechanism mediated by IL-27 might indicate a new mechanism for the chronic pain hypersensitivity.

## Introduction

Chronic pain is defined as pain that lasts over three months. Existing therapeutic strategies for this condition have limited effects^1,2^. Thus, better treatments for chronic pain are eagerly sought. To study the mechanisms that underlie chronic pain, two types of animal models have been widely used: inflammatory and neuropathic pain models^3^. In these models, animals display long-lasting hypersensitivity to thermal and/or mechanical stimuli. Analyses of these models suggest that the immune system plays an important role in the induction of such hypersensitivities. For instance, Interleukin (IL)-17, as well as other pro-inflammatory cytokines including IL-1β, IL-6, tumour necrosis factor (TNF)-α, and interferon (IFN)-γ, have been reported to induce and/or enhance pain behaviour in normal animals. In contrast, IL-10, IL-1ra, IL-4, and IL-13 have been reported to attenuate pain behaviour in animal models of chronic pain diseases^4–8^.

IL-27 is a heterodimeric cytokine consisting of Epstein-Barr virus–induced gene 3 (EBI3) and IL-27p28 subunits. It binds to a receptor comprising gp130 (shared with many cytokine receptors, including IL-6R) and IL-27Rα (also known as WSX-1 or TCCR)^9,10^. IL-27 is mainly produced by antigen presenting cells and is induced by pathogens and immune stimuli^10^. A notable feature of this cytokine is its immunosuppressive and anti-inflammatory roles mediated by the induction of IL-10 and suppression of IL-17 through T cell differentiation. While IL-27 suppresses differentiation from naïve T cells to IL-17-producing T cells (Th17), this cytokine also promotes differentiation from activated T cells to IL-10-producing regulatory T cells (Tr1)^11^.

On examining the role of IL-27 in several different pathological conditions, we have observed that disrupted IL-27/WSX-1 signalling leads to more severe pathological changes, owing to exacerbated inflammation, than those observed in wild-type (WT) animals^12–14^. In addition, IL-27 has demonstrated potential therapeutic action in rheumatoid arthritis^15–17^ and other inflammatory diseases^18–24^. However, the role of IL-27 in pain sensation has not yet been elucidated. The evidence that IL-17 promotes pain while IL-10 has anti-nociceptive effects^4–7,10^ implies that IL-27 may show anti-nociceptive effects.

Here, we report the new distinctive role of IL-27 on the regulation of basal pain sensitivity, which is found unexpectedly. Mice lacking IL-27 subunits (EBI3 or p28) or its receptor (WSX-1) are hypersensitive to several kinds of stimuli applied to the skin. Because we assumed that phenotypic differences would appear after induction of chronic pain due to the inducible nature of IL-27, we did not expect such a hypersensitive phenotype without any treatment. Contrary, the phenotype suggests that IL-27 constitutively controls the threshold of the basal pain sensitivity. In this study, we focus on to investigate the roles of constitutive IL-27 on controlling the basal pain sensitivity.

## Results

### Enhanced pain behaviour in mice lacking IL-27 or its receptor

Mice lacking an IL-27 subunit (EBI3 or p28) or its receptor (WSX-1) appeared healthy and had no obvious abnormalities on visual inspection. Initially, we evaluated locomotor activity and anxiety of the mice, as these factors are known to influence the reliability of assessments of pain behaviours. There were no significant differences among WT, *WSX-1*^−/−^ (WSX-1-deficient), *EBI3*^−/−^ [Epstein-Barr virus-induced gene 3 (EBI3)-deficient)], and *p28*^−/−^ (p28-deficient) mice in any parameters obtained from the open field and rotarod tests, suggesting that IL-27/WSX-1 signalling-deficient mice did not have substantial dysfunctions in locomotor activity or anxiety-like behaviour (see Supplementary Fig. S1).

**Figure 1:**
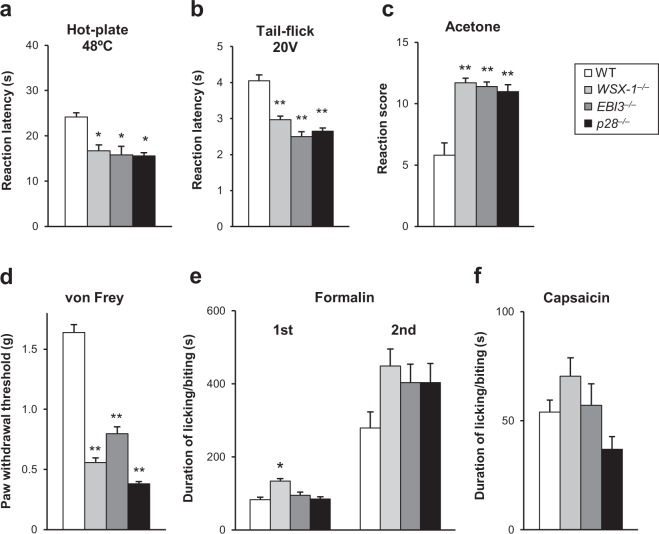
Lack of IL-27/WSX-1 signalling results in enhanced nociceptive behaviours. Reaction latencies to heat measured with the (**a**) hot-plate and (**b**) tail-flick tests. (**c**) Acetone test, in which values represent reaction scores after acetone application to a hind paw. (**d**) Fifty percent paw withdrawal threshold measured with the von Frey test. (**e**) Duration of licking and biting during the first (0–10 min) and second (10–60 min) phases after formalin injection into a hind paw. (**f**) Duration of licking and biting after capsaicin injection into a hind paw. *n* = 5–10. *P < 0.05, **P < 0.01 vs. WT mice. Kruskal-Wallis test with Steel-Dwass test. All data are expressed as mean ± SEM.

To test whether the lack of IL-27/WSX-1 signalling would affect pain behaviour, we measured responses evoked by heat, cold, or mechanical stimuli in *WSX-1*^−/−^, *EBI3*^−/−^, *p28*^−/−^, and WT mice. Reaction latencies measured with the hot-plate test (48 °C) in *WSX-1*^−/−^, *EBI3*^−/−^, and *p28*^−/−^ mice were significantly reduced compared to the latency in WT mice (Fig. 1a). Sensitivity to noxious heat was also evaluated with the tail-flick test. Consistent with the hot-plate test results, tail-flick latencies in *WSX-1*^−/−^, *EBI3*^−/−^, and *p28*^−/−^ mice^−^ mice were significantly reduced compared to the latency in WT mice (Fig. 1b).

We used the acetone test to investigate sensitivity to cold stimuli. In this test, reaction scores in *WSX-1*^−/−^, *EBI3*^−/−^, and *p28*^−/−^ mice were significantly higher than the score in WT controls (Fig. 1c). In a test of mechanical threshold using von Frey filaments, paw withdrawal thresholds in *WSX-1*^−/−^, *EBI3*^−/−^, and *p28*^−/−^ mice were significantly lower than the threshold in WT controls (Fig. 1d).

We also tested responses to chemical stimuli by injecting formalin or capsaicin into the hind paws of WT, *WSX-1*^−/−^, *EBI3*^−/−^, and *p28*^−/−^ mice. In the first phase (0–10 min) of the formalin test, the duration of licking and biting time in *WSX-1*^−/−^ but not *EBI3*^−/−^ or *p28*^−/−^ mice was slightly but significantly increased compared with that in WT controls (Fig. 1e). In the second phase (10–60 min) of the same test, the duration of licking and biting time tended to increase in *WSX-1*^−/−^, *EBI3*^−/−^, and *p28*^−/−^ mice compared with that in WT controls. However, no significant differences were detected (Fig. 1e). In the capsaicin test, no significant differences were detected among WT, *WSX-1*^−/−^, *EBI3*^−/−^, and *p28*^−/−^ mice (Fig. 1f). These data suggest that mice lacking IL-27/WSX-1 signalling show increased sensitivity to noxious heat and cold stimuli, as well as mechanical stimuli, without any prior treatment.

### IL-27 constitutively controls threshold of sensation

We next examined whether deletion of *WSX-1*, *EBI3*, or *p28* resulted in anatomical abnormality of the spinal dorsal horn or primary afferents. We evaluated sections from the spinal cord (SC) and dorsal root ganglia (DRG) of WT, *WSX-1*^−/−^, *EBI3*^−/−^, and *p28*^−/−^ mice. We used NF200 (neurofilament 200), CGRP (calcitonin gene-related peptide), and IB4 (isolectin B4) as markers for myelinated fibres, peptidergic C-fibres, and non-peptidergic C-fibres, respectively^25,26^. In both the DRG and spinal dorsal horn (central terminal region of these afferents), the distribution of these markers was comparable among WT, *WSX-1*^−/−^, *EBI3*^−/−^, and *p28*^−/−^ mice (see Supplementary Fig. S2). Furthermore, we measured the mRNA expression of ion channels (transient receptor potential [TRP], Piezo, and P2XR channels), which are known to be involved in pain and tactile sensation, in DRG and/or skin tissues obtained from WT and IL-27/WSX-1 signalling-deficient mice. The expression of these channels appeared to be normal in mice lacking IL-27/WSX-1 signalling (see Supplementary Fig. S3). We concluded that there were no detectable abnormalities in the expression of anatomical markers and mRNA encoding major ion channels in the spinal dorsal horn, DRG, or footpad skin of mice lacking IL-27/WSX-1 signalling.

Next, we investigated the possible involvement of spinal glial cells, because several lines of evidence have shown that spinal glial cells regulate pain sensitivity^27–29^. The number of Iba1 (ionised calcium-binding adapter molecule-1)-positive microglia as well as SOX9 [SRY (sex determining region Y)-box 9]-positive astrocytes in the spinal dorsal horn of WT, *WSX-1*^−/−^, *EBI3*^−/−^, and *p28*^−/−^ mice were comparable, with no significant differences. Microglial cells with ramified morphology indicating the ‘resting form’ were the majority in the spinal sections from WT mice as well as IL-27/WSX-1 signalling-deficient mice. This suggests that activation of spinal microglia is not involved in thermal/mechanical hypersensitivity (see Supplementary Fig. S4a–d). We further confirmed that paw withdrawal thresholds in naïve WT, *WSX-1*^−/−^, *EBI3*^−/−^, and *p28*^−/−^ mice were insensitive to minocycline (50 mg/kg), a microglial inhibitor, while the same treatment significantly reversed hypersensitivity induced by spinal nerve injury in WT mice, as previously reported^30^ (see Supplementary Fig. S4e). We therefore concluded that basal activity of spinal microglia in *WSX-1*^−/−^, *EBI3*^−/−^, and *p28*^−/−^ mice made little, if any, contribution to their hypersensitive phenotypes.

**Figure 2:**
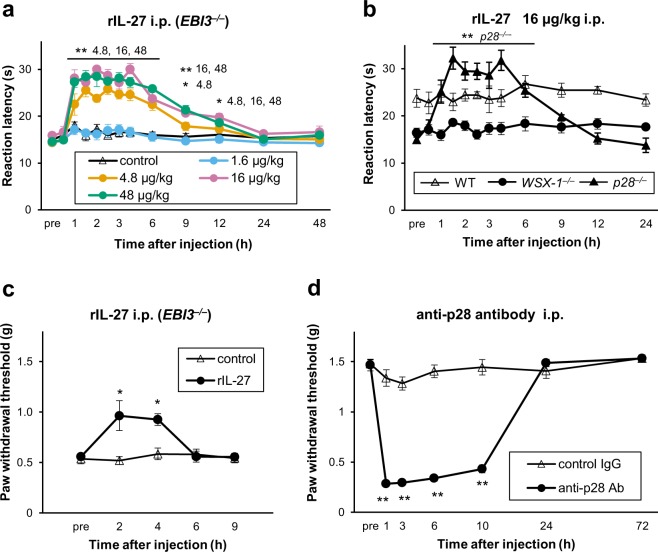
IL-27 is an endogenous regulator of thermal and mechanical nociceptive responses. (**a**) Single i.p. injection of rIL-27 dose-dependently prolonged hot-plate latency in *EBI3*^−/−^ mice. (**b**) Quick restoration of hot-plate latencies in *p28*^−/−^, but not *WSX-1*^−/−^ and WT mice following a single i.p. injection of rIL-27 (16 µg/kg). (**c**) A single i.p. injection of rIL-27 (16 µg/kg) also significantly alleviated mechanical hypersensitivity in *EBI3*^−/−^ mice. (**d**) A single i.p. injection of neutralising antibody against p28 (1.6 mg/kg) decreased the paw withdrawal threshold in naïve WT mice. *n* = 5 animals/group. *P < 0.05, **P < 0.01 vs. (**a** and **c**) saline control, (**b**) pre (before injection) or (**d**) control antibody. (**a**,**c**, and **d**) Two-way ANOVA followed by Tukey’s *post hoc* tests or (**b**) Steel-Dwass test. All data are expressed as means ± SEM.

Next, we investigated whether the basal hypersensitivity observed in mice lacking IL-27/WSX-1 signalling could be reversed by restoration of IL-27. The hot-plate test revealed that a single i.p. injection of rIL-27 extended paw withdrawal latency in *EBI3*^−/−^ mice in a dose-dependent manner (Fig. 2a). Injection of rIL-27 (16 µg/kg) also increased the hot-plate latency in *p28*^−/−^ but not in *WSX-1*^−/−^ mice, suggesting that the effect of rIL-27 depended on its specific receptor, WSX-1 (Fig. 2b). In contrast, WT mice did not show any changes in paw withdrawal latency even after injection of rIL-27 at a higher dose (48 µg/kg) (see Supplementary Fig. S5). Partial but significant recovery of the paw withdrawal threshold for mechanical stimuli was also observed in *EBI3*^−/−^ mice after rIL-27 (16 µg/kg) injection (Fig. 2c). These results indicate that under normal conditions endogenous IL-27 regulates the threshold of cutaneous sensation and that the hypersensitive phenotype of knock-out mice do not result from irreversible developmental abnormalities.

To further confirm this conclusion, we administered an i.p. injection of a neutralising antibody against p28 (1.6 mg/kg) into WT mice and assessed the mechanical threshold with the von Frey test. A single i.p. injection of anti-p28 antibody induced long-lasting mechanical hypersensitivity (Fig. 2d). These findings strongly suggest that in normal conditions constitutive expression of IL-27 plays an important role in controlling pain sensitivity.

### Possible involvement of a novel mechanism for hypersensitivity observed in mice lacking IL-27/WSX-1 signalling

**Figure 3:**
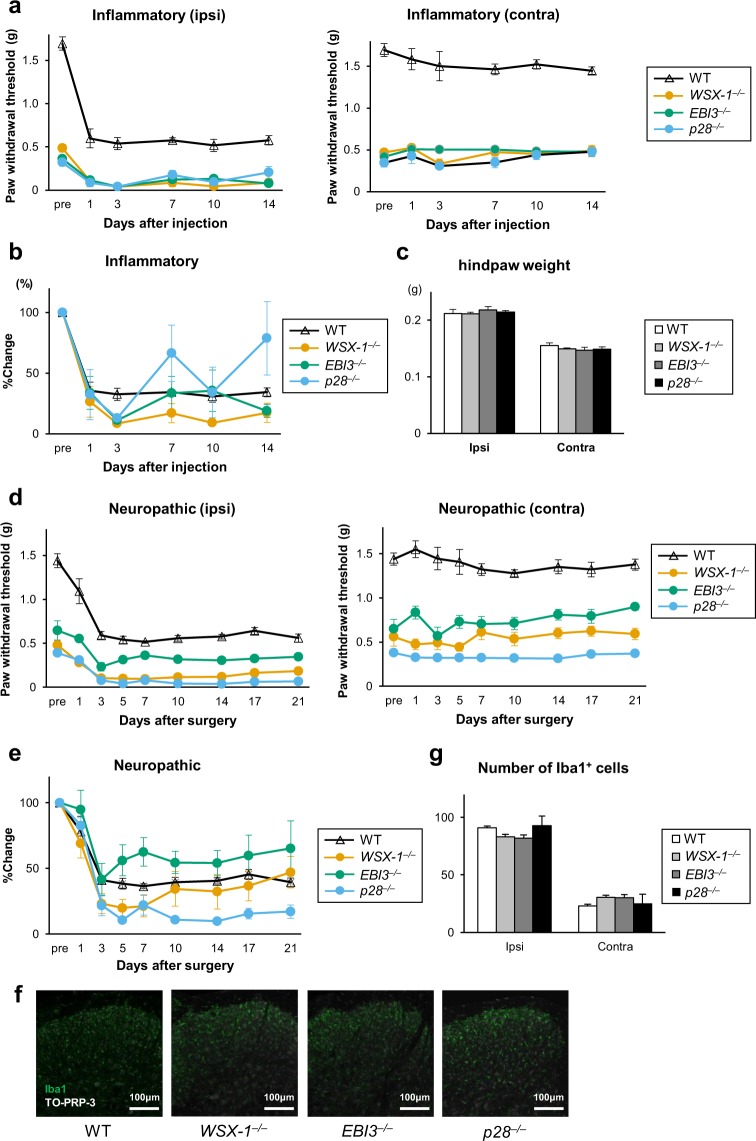
Mice lacking IL-27/WSX-1 signalling still develop mechanical hypersensitivity after inflammation and peripheral nerve injury. Mice were subjected to the (**a**–**c)** inflammatory or (**d**–**g**) neuropathic pain model. Fifty percent paw withdrawal threshold before and after (**a**) CFA injection or (**d**) spinal nerve injury. (**b**,**e**) The percent change in paw withdrawal threshold calculated from a and d, respectively. Each value after CFA injection or nerve injury was normalised by the control value (pre) in each mouse. (**c**) Hind paw weight, as a measure of oedema, in mice 14 days after i.pl. CFA injection. (**f**) Representative immunofluorescence labelling for Iba1 (green) with nuclear staining with TO-PRO-3 (blue) in a transverse section of the dorsal horns from the L4 segment of WT, *WSX-1*^−/−^, *EBI3*^−/−^, and *p28*^−/−^ mice 7 days after spinal nerve injury. (**g**) Number of Iba1-positive cells in the ipsilateral or contralateral dorsal horn of L4 spinal segments dissected from WT, *WSX-1*^−/−^, *EBI3*^−/−^, and *p28*^−/−^ mice 7 days after spinal nerve injury. *n* = 5 animals/group. All data are expressed as means ± SEM.

To explore the mechanisms underlying chronic hypersensitivity in mice lacking IL-27/WSX-1 signalling, we first investigated whether there were commonalities with mechanisms underlying known chronic pain models for inflammatory or neuropathic pain. If the hypersensitive phenotype of mice lacking IL-27/WSX-1 signalling is indeed a result of constitutive activation of mechanism(s) involved in other chronic pain models, further enhancement of pain behaviour on subjecting the mice to treatment leading to induction of other chronic pain models should not be expected as the inducible pathways are already engaged in processing pain information. First, we tested the inflammatory pain model. To produce persistent inflammatory pain, complete Freund’s adjuvant (CFA) was injected into the plantar surface of the left hind paw. The von Frey test revealed that both WT and IL-27/WSX-1 signalling-deficient mice developed significant mechanical hypersensitivity after CFA treatment, without any effect on the contralateral side (Fig. 3a). Differences at baseline between WT and IL-27/WSX-1 signalling-deficient mice made it difficult to compare the degree of changes in the paw withdrawal threshold after the treatment. Hence, we also calculated the percent changes to compare the degree of differences in hypersensitivity indicated by paw withdrawal threshold change from baseline among WT, *WSX-1*^−/−^, *EBI3*^−/−^, and *p28*^−/−^ mice. The percent changes largely overlapped and were indistinguishable among the groups (Fig. 3b). In addition, we confirmed that induced behavioural hypersensitivities in IL-27/WSX-1 signalling-deficient mice were not affected by stimulus intensity that reflect the degree of inflammation. There were no significant differences in swelling of the CFA-injected hind paw among WT, *WSX-1*^−/−^, *EBI3*^−/−^, and *p28*^−/−^ mice (Fig. 3c). These results indicate that the mechanism underlying basal hypersensitivities observed in naïve IL-27/WSX-1 signalling-deficient mice is not the same as the mechanism underlying the inflammatory pain model.

Next, we tested the neuropathic pain model. To produce peripheral neuropathic pain, the left L4 spinal nerve was injured. As in the case of CFA injection, induction of mechanical hypersensitivity was observed in both WT and IL-27/WSX-1 signalling-deficient mice (Fig. 3d). The percent changes largely overlapped and were indistinguishable among the groups (Fig. 3e). In addition, spinal microgliosis appeared to be unaffected in IL-27/WSX-1 signalling-deficient mice, because there were no significant differences in the number of microglia in the spinal dorsal horn among these mouse lines (Fig. 3f,g). The observation of microgliosis in the deficient mice was consistent with induced hypersensitive behaviour of these mice after nerve injury, because microglial proliferation is known to contribute, at least in part, to the mechanisms involved in neuropathic pain. These results suggest that the mechanism underlying the basal hypersensitivities observed in naïve IL-27/WSX-1 signalling-deficient mice is not the same as the mechanism underlying the neuropathic pain model.

Taken together, the results suggest that the basal hypersensitivities in mice lacking IL-27/WSX-1 signalling are likely induced by a mechanism that differs and is independent from those underlying the inflammatory and neuropathic pain models. Furthermore, the evidence that the induced hypersensitivity was added to the basal hypersensitivity in the mutant mice suggests that the basal hypersensitivity exist persistently, parallelly, and independently after induction of chronic pain, i.e. in pathological conditions. Thus, we concluded that the chronic hypersensitivities observed in mice without IL-27/WSX-1 signalling were induced by a novel mechanism.

### Lack of IL-27/WSX-1 signalling also facilitated neuronal activity recorded in skin-nerve preparations

As described above, peripherally administered rIL-27 resulted in reversal of the hypersensitive phenotypes in IL-27-deficient mice, while the same treatment with IL-27 neutralising antibody resulted in induction of hypersensitivity in WT mice. Thus, we speculated that IL-27 binds to receptors expressed in peripheral tissues, because molecules such as cytokines or antibodies are thought to be too large to penetrate the blood-brain barrier. Therefore, we tested whether the hyperactive phenotype in mice lacking IL-27/WSX-1 signalling could be reproduced in a skin-nerve preparation. With this preparation, we could record the primary afferent activity when the skin was stimulated.

**Figure 4:**
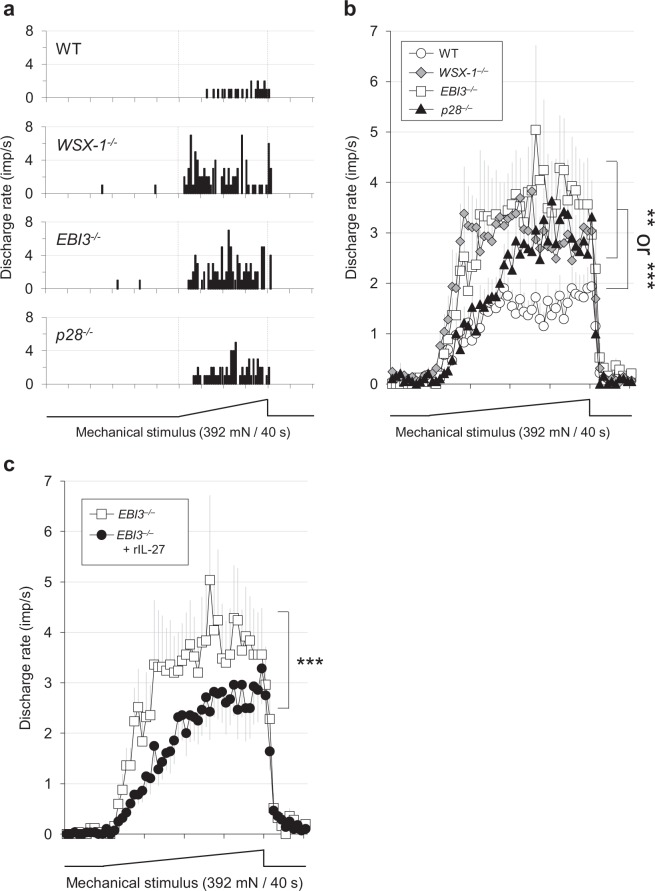
Facilitated mechanical sensitivity of C-fibre nociceptors in mice lacking IL-27/WSX-1 signalling. (**a**) Sample recordings of peri-stimulus time histogram. Abscissa: time in seconds. Ordinate: discharge rate of C-nociceptors. Lowest trace: force output of the mechanical stimulus. (**b**) Summarised mechanical response patterns. The response magnitude was significantly greater in *WSX-1*^−/−^, *EBI3*^−/−^, and *p28*^−/−^ mice compared with WT. (**a**,**b**) WT (n = 28), *WSX-1*^−/−^ (n = 29), *EBI3*^−/−^ (n = 25), *p28*^−/−^ (n = 19). *P* < 0.01–0.001. Kruskal-Wallis test followed by Dunn’s multiple comparison test. (**c**) Reduced mechanical response magnitude in *EBI3*^−/−^ mice treated with rIL-27 compared with non-treated *EBI3*^−/−^ mice. *P* < 0.001. Mann-Whitney U test. The data for non-treated *EBI3*^−/−^ mice in panel **c** are identical to those of the *EBI3*^−/−^ mice in panel b.

Single-unit recordings from C-fibre nociceptors of WT, *WSX-1*^−/−^, *EBI3*^−/−^, and *p28*^−/−^ mice showed comparable values for general characteristics, such as conduction velocity, background discharge, and size of receptive field (see Supplementary Fig. S6). However, mechanical stimulation of the skin revealed that the discharge rate of C-fibres from *WSX-1*^−/−^, *EBI3*^−/−^, and *p28*^−/−^ mice was significantly higher than that of WT mice. This indicates that the basal hypersensitivity in mice lacking IL-27/WSX-1 signalling is replicated in the *in vitro* preparation (Fig. 4a,b). Furthermore, the discharge rate of C-fibres recorded from rIL-27 treated *EBI3*^−/−^ mice was significantly lower than that recorded from non-treated *EBI3*^−/−^ mice (Fig. 4c). These results indicate that a potential site of action for IL-27 lies within the tissues used for the skin-nerve preparations.

### Local IL-27 controls mechanical sensitivity

**Figure 5:**
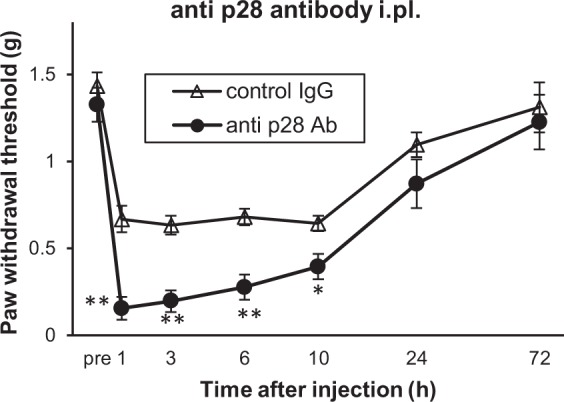
Intraplantar blockade of IL-27 decreased paw withdrawal threshold in naïve WT mice. Fifty percent paw withdrawal threshold before and after i.pl. injection of a neutralising antibody against p28 or control IgG into WT mice. *n* = 3–5 animals/group. *P < 0.05, **P < 0.01 vs. control IgG. Two-way ANOVA followed by Tukey’s *post hoc* tests. All data are expressed as means ± SEM.

Given that the results of the experiments using the skin-nerve preparation suggested that a possible site of action for IL-27 might be included within the tissue used for the preparation, we investigated whether local injection of a neutralising antibody against p28 could reproduce the hypersensitivity in WT mice that was observed with systemic (i.p.) injection. Local [intraplantar (i.pl.)] injection of the neutralising antibody against p28 (16 µg/kg) significantly reduced the paw withdrawal threshold compared with a control antibody (Fig. 5). Together with the results from the skin-nerve preparations, these results provide further support for the suggestion that a potential site of action for IL-27 is included within the footpad.

Furthermore, we attempted to identify cell populations expressing WSX-1, in several tissues, especially those forming a part of the pain pathway, such as the SC, DRG, and footpad skin. With our real-time PCR and *in situ* hybridisation strategies, we could detect substantial expression of *WSX-1* mRNA in the spleen and thymus as positive controls. On the other hand, the same method revealed extremely low or undetectable expression of this gene in the SC, DRG, and footpad skin (see Supplementary Fig. S7). We conclude that IL-27 probably acted locally on cells located within the skin such as that of the footpad but the expression of *WSX-1* mRNA was presumably maintained at extremely low levels.

## Discussion

The main findings of this study were: (1) *WSX-1*^−/−^, *EBI3*^−/−^, and *p28*^−/−^ mice were extremely hypersensitive to heat, cold, and mechanical stimuli (Fig. 1a–d); (2) restoration of IL-27 in *EBI3*^−/−^ and *p28*^−/−^ mice rapidly ameliorated these hypersensitivities, while blockade of IL-27 in WT mice resulted in rapid induction of hypersensitivity (Fig. 2); (3) application of established chronic pain models to mice lacking IL-27/WSX-1 signalling further enhanced the basal hypersensitivity (Fig. 3a,d); and (4) discharge rates recorded with *in vitro* skin-nerve preparations from IL-27/WSX-1 signalling-deficient mice were higher than those from WT mice (Fig. 4a,b). These results indicate that IL-27 is constitutively produced and is required to regulate thermal and mechanical sensation. Furthermore, we suggest that the mechanism underlying this phenomenon is different and independent from those underlying established chronic pain models. The summation of basal and induced hypersensitivity in the mutant mice strongly suggests persistent, parallel, and independent existence of the basal hypersensitivity after induction of chronic pain, i.e. in pathological conditions. Thus, the major role of IL-27 likely contributes to controlling the basal sensitivity rather than to being involved in the pathological changes. The mechanism may provide a novel method for controlling pain.

The rapid effects of rIL-27 in IL-27 subunit-deficient mice and IL-27 neutralising antibody in WT mice (Fig. 2) indicate that the hypersensitive phenotype depends on absence of IL-27 but not developmental abnormality. Our histochemical analysis showed normal distributions of markers for the different classes of primary afferents in spinal dorsal horn and DRG of mice lacking IL-27/WSX-1 signalling and our real-time PCR analysis also revealed normal expression of ion channels including TRP, Piezo, and P2XR, which are known to be involved in pain and tactile sensation in the DRG and skin tissues in these mice (see Supplementary Fig. S3). Together with the rapid effects described above, these results indicate that the role of IL-27 in cutaneous sensitivity is likely not mediated by altered anatomical structure and/or gene expression of these ion channels. Rather, IL-27 seems to directly affect cells that are involved in the pain pathway. In addition, the rapid effects described above also indicate that the actions of IL-27 are not mediated by T cell differentiation. Thus, unlike other pathological conditions reported^15,21–24^, the role of IL-27 on controlling basal pain sensitivity seems not to be mediated by T cells producing IL-17 or IL-10, unexpectedly.

Our results strongly indicate that IL-27 constitutively controls pain sensitivity. It is well known that IL-27 is mainly produced by the antigen presenting cells, such as dendritic cells (DCs) and macrophages^10^. The expression of IL-27 depends on the expression of p28 because of its limited expression compared to EBI3. The expression of p28 is induced by activation of Toll-like receptors, costimulatory molecules such as CD40 and CD137, and type I and II interferon signalling^10^. However, little is known about constitutive activity of IL-27/WSX-1 signalling in the normal condition. By using cDNA libraries of various tissues or cells from human or mouse, expression of p28 and EBI3 has been examined^31^. Among human foetal tissues (spleen, testis, uterus, ovary, brain, lung, and kidney) and placenta (28 wk), the expression of EBI3 is found in the placenta and spleen and that of p28 is found in the placenta. Among the mouse tissues tested (colon, lymph nodes, peyer’s patches, stomach, lung, spleen, spinal cord, skin, pancreas, kidney, and thymus), the expression of EBI3 is found in all the tissues tested although there are differences in its expression level, and that of p28 is found in the thymus and the spleen. In cDNA libraries of human and mouse cells that include endothelial cells (ECs), DCs, macrophages, monocytes, hematopoietic precursors, natural killer (NK) cells, B cells, splenocytes, T cells, and fibroblasts, clear expression of both p28 and EBI3 is detected in monocytes, macrophages, and DCs. Most of these cells are ‘activated’, while some of these are ‘resting’. In other cell types, EBI3 expression is found widely across cell types, while p28 expression is very limited. Thus, IL-27 may be supplied constitutively by certain cells in the normal condition. Alternatively, IL-27 may be induced by constitutive activation by certain factors. A recent investigation has shown that a subset of DCs in the skin is constitutively exposed to commensals. This interaction is important to control skin immunity appropriately but not to be associated with inflammation^32^. Similar constitutive stimuli may be involved in the spontaneous IL-27 supply.

Regarding the receptors for IL-27, because the expression of gp130 is found in almost all organs, the expression of the IL-27 receptor depends on the expression of WSX-1. The expression of WSX-1 mRNA has been detected by northern blot hybridization in various human tissues include spleen, thymus, peripheral blood leukocyte, lymph node, heart, lung, small intestine, thyroid, liver, skeletal muscle, kidney, pancreas, prostate, testis, ovary, spinal cord, trachea, and bone marrow^33^, and by the quantitative PCR in human cDNA libraries from various cell types including immune cells such as monocyte, DCs, T cells, B cells, neutrophils, mast cells, and NK cells, and also other cells such as keratinocytes, smooth muscle, and ECs^9^. Thus, IL-27 receptors are likely expressed in variety of cells in normal condition.

Regarding other interleukins, information of their constitutive activities in pain behaviour is hardly found. IL-1 receptor (IL-1r)-deficient mice show reduced basal sensitivity to thermal and mechanical stimuli^34^, however, the phenotype seems result from developmental changes rather than lack of constitutive IL-1, because acute administration of IL-1ra (receptor antagonist) to WT mice has no effect on basal pain sensitivity. IL-6-dificient mice also show reduced basal sensitivity to thermal and mechanical stimuli^35^, however, the role of constitutive IL-6 on the phenotype is unclear, because the effects of acute supplementation of IL-6 to IL-6-deficient mice or acute blockade of IL-6 in WT mice are not tested. By contrast, mice lacking anti-inflammatory cytokine IL-4 show basal mechanical hypersensitivity^36^. However, the role of constitutive IL-4 on the phenotype is unclear, because of the same reason mentioned about IL-6-deficient mice. In addition, mice lacking IL-10^37^ or IL-17^4^ show no difference in the basal pain threshold compared to WT mice, consistent with our notion that the basal hypersensitivity of IL-27/WSX-1 signalling-deficient mice seems not to be mediated by T cell differentiation to produce these cytokines.

To identify the tissues and cell populations expressing WSX-1 in the pain pathway, mRNA encoding this protein was assayed by using real-time PCR and *in situ* hybridisation, but these attempts resulted in revealing extremely low or undetectable expression of *WSX-1* mRNA in the SC, DRG, and footpad skin (see Supplementary Fig. S7). Thus, the cell populations responsible for IL-27 action in this phenomenon remain unknown. However, the current results suggest that IL-27 affects peripheral tissues rather than the central nervous system, because (1) rIL-27 or IL-27 neutralising antibody injected intraperitoneally cannot penetrate the brain through the blood-brain barrier; (2) i.pl. application of IL-27 neutralising antibody increased pain behaviour (Fig. 5); and (3) data recorded from the *in vitro* skin-nerve preparations was consistent with the behavioural phenotype, i.e. cells responding to IL-27 possibly exist within these tissues (peripheral nerves and/or cells in the skin).

In the peripheral tissues, one possible target of IL-27 would be the primary afferents. It has been reported that Nav1.8-expressing C-fibres contain gp130 and respond to a fusion protein comprising IL-6 and the soluble type IL-6 receptor, which is known to stimulate gp130 without membrane-bound type IL-6 receptors^38^. However, several observations suggest that IL-27 does not directly affect C-fibre nociceptors: (1) *WSX-1* mRNA in the DRG was undetectable with *in situ* hybridisation and was estimated to be extremely low with quantitative PCR (see Supplementary Fig. S7); (2) direct stimulation of TRPV1-expressing C-fibres by capsaicin did not show any significantly different effects between WT and IL-27/WSX-1 signalling-deficient mice in the behavioural test (Figs. 1f); and (3) in our preliminary experiments, capsaicin-evoked calcium influx in DRG neurons was comparable between WT and *WSX-1*^−/−^ mice (data not shown). Furthermore, pain-related responses of *EBI3*^−/−^ and *p28*^−/−^ mice in the first phase of the formalin test, in which TRPA1-expressing C-fibres play a crucial role^39^, were also comparable to that of WT mice (Fig. 1e). In the case of *WSX-1*^−/−^ mice, the increase in responses was significantly higher than that of WT mice (Fig. 1e) for unknown reasons. Although this inconsistency is difficult to interpret and requires further analysis, it suggests that hypersensitive phenotypes of at least *EBI3*^−/−^ and *p28*^−/−^ mice are not due to direct activation of TRPA1-expressing C-fibres. Taken together, these results suggest that IL-27 controls C-fibre firing indirectly, via other cells expressing WSX-1.

We speculate that the cell types responsible for this phenomenon lie within the skin because of the reasons mentioned above. Within the skin, keratinocytes and immune cells such as DCs are likely good candidates, because WSX-1 expression is detected in the cDNA libraries of these cells^9^. Furthermore, the evidences that these cells are able to respond to IL-27 strongly suggest that these cells express functional IL-27 receptors^40,41^. In our current data, the expression of WSX-1 mRNA in the skin was detected by real-time PCR at very low level (see Supplementary Fig. S7a), probably because we used RNA samples prepared form the skin tissue but not from purified cells. Thus, it is very likely that functional IL-27 receptors are expressed in the skin at least in keratinocytes and DCs, while the expression level seems to be as low as detection limit or below for *in situ* hybridization.

A recent report has shown a clear interaction between dermal DCs and primary afferents, especially C-fibre nociceptors^42^. Thus, it is possible that IL-27 somehow controls these interactions through DCs. Alternatively, this may occur via keratinocytes. Keratinocytes are believed to respond to thermal and mechanical stimulation^43,44^, and are known to release pain-related substances such as adenosine triphosphate (ATP) and opioids^45,46^. A recent optogenetic study reported that selective activation of keratinocytes can evoke pain-related behaviour^47^. Another recent study also demonstrate ATP released from keratinocyte mediate touch^48^. Therefore, the available evidence suggests that the effects of IL-27 might be mediated by keratinocytes, although data supporting this notion are still lacking.

We tested the possible involvement of increased ATP or decreased opioids in the hypersensitivities of IL-27/WSX-1 signalling-deficient mice, because these are known to be released from keratinocytes. However, the non-selective P2 receptor antagonist PPADS (pyridoxal-phosphate-6-azo (benzene-2,4-disulfonic acid) failed to suppress mechanical hypersensitivity in *WSX-1*^−/−^ and *p28*^−/−^mice, suggesting that ATP is unlikely to be involved in the basal hypersensitivity of IL-27/WSX-1 signalling-deficient mice. Moreover, the non-selective opioid receptor antagonist naloxone did not block the anti-hyperalgesic effects of rIL-27 in *EBI3*^−/−^ mice (see Supplementary Fig. S8). Thus, the opioid systems are unlikely to be controlled by IL-27/WSX-1 signalling. In addition, the chronic pain-like phenotype of IL-27/WSX-1 signalling-deficient mice could be treated with opioid agonists (but not ibuprofen) and the expression of opioid receptors was preserved in these mutant mice (see Supplementary Figs S9 and S10), indicating that the lack of inhibition by naloxone after rIL-27 injection in *EBI3*^−/−^ mice is unlikely due to breakdown of the opioid system. Further studies, such as cell-specific deletion of IL-27 subunits or WSX-1, are required to obtain useful information about the underlying mechanism of IL-27-induced anti-hyperalgesic action.

In summary, our results clearly show that constitutive expression of IL-27 controls thermal and mechanical sensation. In the absence of IL-27, mice develop hypersensitivity to heat, cold, and mechanical stimuli. Thus, IL-27 plays an important role in controlling cutaneous responsiveness to thermal and mechanical stimuli. We suggest that the mechanisms underlying hypersensitivity induced by IL-27 blockade are different from those of the established chronic pain models. Exploring IL-27 signalling may provide new insights into the mechanisms of pain-hypersensitivity.

## Methods

### Animals

All animal experiments were conducted according to the ‘Fundamental Guidelines for Proper Conduct of Animal Experiments and Related Activities in Academic Research Institutions’ (Ministry of Education, Culture, Sports, Science and Technology of Japan) and the institutional guideline (the ethical guidelines of Saga University or the Regulations for Animal Experiments at Nagoya University). They were also in accordance with the ethical guidelines of the International Association for the Study of Pain^49^. All procedures in animal experiments were approved by the Saga University Animal Care and Ethical Use Committee (#28-001-0) or the Institutional Animal Care and Use Committee of the Research Institute of Environmental Medicine, Nagoya University (#15197).

All mice used in this study were males aged between 8 and 13 weeks (weights, 22–28 g) at the start of each experiment. WSX-1-deficient mice (*WSX-1*^−/−^), EBI3-deficient mice (*EBI3*^−/−^), and p28-deficient mice (*p28*^−/−^) mice were generated and the lack of WSX-1, EBI3, and p28 proteins in corresponding mutant mice were verified as previously described^50–52^. They were backcrossed at least 10 times to C57BL/6 mice (Clea Japan, Inc, Tokyo, Japan), which were used as the corresponding controls. Mice were housed in groups of 2–5 per cage at a temperature of 23.0 ± 2 °C and humidity of 55 ± 5%, with an automatic 12-hr light-dark cycle (lights on 8 AM to 8 PM). The mice had access to food and water *ad libitum*.

### Behavioural assessments

#### Thermal sensitivity

To assess sensitivity to heat, we conducted the hot-plate and tail-flick tests. In the hot-plate test, mice were allowed to habituate to the hot-plate testing apparatus (MK-350HC, Muromachi Kikai, Tokyo, Japan) for 3 min before data collection. The mice were then placed on an electrically heated metal plate maintained at 48 ± 0.3 °C. The hot-plate latency was measured as the latency to lick or bite their hind paws or to jump, with a maximal cut-off time of 60 s to prevent tissue damage.

Noxious heat-evoked tail-flick responses were detected with the application of radiant heat (Tail-Flick Unit 7360, Ugo Basile, Italy) to the tails of mice under sevoflurane (2.0–2.5%) anaesthesia. The intensity of the heat stimulus was adjusted to 20 V, with a cut-off time of 10 s.

To assess cold sensitivity^53,54^, a drop (50 µl) of acetone (Wako Pure Chemical Industries, Ltd., Osaka, Japan) was applied to the hind paw of a mouse on a wire mesh. The response evoked by evaporative cooling was monitored, and scaled (0, no response; 1, quick withdrawal, flick or stamp of the paw; 2, prolonged withdrawal or repeated flicking (more than 2 times) of the paw; 3, repeated flicking of the paw with licking directed at the plantar surface of the hind paw) according to a previous study^53^. Acetone was applied five times alternately to each hind paw and cumulative scores were generated for each mouse.

#### Mechanical sensitivity

To assess mechanical sensitivity, mice were placed individually in a transparent glass cylinder and separated from each other by an opaque divider, which was placed on a wire mesh. They were allowed to habituate to the test apparatus for at least 1 h prior to data collection. Subsequently, calibrated von Frey filaments (0.02–2.0 g, Stoelting Co., Wood Dale, IL) were applied to the mid-plantar surfaces of the hind paws until the mice withdrew their paws in response to the mechanical stimuli. The 50% paw withdrawal threshold was determined with the up-down method^55^.

#### Formalin-induced nociceptive behaviours

The procedure used for the formalin test has been described elsewhere^54,56,57^. Mice were acclimatised to the test apparatus for at least 30 min prior to data collection. To assess formalin-induced nociceptive behaviour, formalin (5%, 10 µl/paw, Wako) was injected into the plantar surface of the right hind paw of each mouse with a 30-gauge needle connected to a microsyringe. Following the injection, the mice were placed individually in a clear glass cylinder (110 × 150 mm). The duration of licking and biting responses to the injected hind paw was then measured as the nociceptive response, for 60 min at 5 min intervals.

An initial acute phase (first phase, during the first 10 min after the formalin injection) was followed by a relatively short quiescent period, and then by a prolonged tonic response (second phase, beginning 10 min after the formalin injection). The first phase is presumed to result from a direct activation of the primary afferent nociceptors, and the second phase involves peripheral inflammatory events and central sensitisation^58^. The cumulative times spent engaged in nociceptive behaviours during the first and second phases were analysed separately.

#### Capsaicin-induced nociceptive behaviours

The capsaicin test was performed according to previous studies^59,60^. Mice were habituated to the test apparatus for 10 min prior to data collection. In the tests of capsaicin-induced pain, mice were injected in the plantar surface of the right hind paw with 20 µl of capsaicin (1.6 µg/paw, Wako) with a 30-gauge needle connected to a microsyringe. Immediately after the injection, the mice were placed individually in a clear glass cylinder (90 × 120 mm), and the duration of licking and biting responses to the injected hind paw was measured for 5 min as an indicator of the nociceptive responses. The capsaicin was first dissolved in dimethyl sulfoxide (DMSO) and then diluted in saline (1:100).

#### Chronic pain models

For the inflammatory pain model, CFA (0.01 mg/20 µl, BD, Franklin Lakes, NJ) was injected into the plantar surface of the right hind paw with a 26-gauge needle^54^. The von Frey test was used to measure the 50% withdrawal threshold, and data were collected before and after the injection. For measurement of the hind paw swelling induced by CFA injection, the weights of the right (CFA) and left [phosphate-buffered saline (PBS), control] hind paws, amputated at the ankle, were measured 14 days after the injection.

For the neuropathic pain model, we used the spinal nerve injury model^61^ with some modifications^54^. Briefly, the left fourth lumbar spinal nerve of mice was transected under isoflurane (2%) anaesthesia. We determined the 50% withdrawal threshold with the von Frey test, and data were collected before and after peripheral nerve injury. In another set of experiments, mice from each strain were used for immunohistochemistry to count the microglia in the spinal dorsal horn 7 days after injury.

#### Statistical analyses for behavioural assessments

Data are expressed as means ± SEM. Statistical significance was evaluated with the Kruskal-Wallis test with Steel-Dwass *post hoc* tests or two-way ANOVA followed by Tukey’s *post hoc* tests. *P* < 0.05 was considered statistically significant.

### Drug application

To investigate whether rIL-27 could reverse the hypersensitive phenotypes observed in mice lacking IL-27 signalling, *EBI3*^−/−^ mice received an i.p. injection of rIL-27 (1.6, 4.8, 16, and 48 µg/kg, BioLegend, San Diego, CA) or saline. *WSX-1*^−/−^, *p28*^−/−^, and WT mice also received an i.p. injection of rIL-27 (16 µg/kg). The hot-plate test (at 48 °C) was used to evaluate the effects of rIL-27 on thermal sensitivity. In another set of experiments, *EBI3*^−/−^ mice received an i.p. injection of rIL-27 (16 µg/kg) or saline to assess the effects on mechanical sensitivity by using the von Frey test.

To test whether neutralising antibodies against IL-27 could mimic phenotypes observed in mice lacking IL-27/WSX-1 signalling, WT mice received an i.p. or i.pl. injection of anti-mouse IL-27 (p28) neutralising antibody (1.6 mg/kg or 16 µg/kg, respectively, mouse IgG2a κ isotype, Affymetrix, Santa Clara, CA) or the same dose of an isotype-matched control antibody (mouse IgG2a κ isotype, Affymetrix). The von Frey test was used to evaluate the effects of antibodies. The dose of antibody used for systemic injection was virtually the same or lower compared with those in previous studies^62–64^.

### Immunohistochemistry

Seven days after spinal nerve injury, *WSX-1*^−/−^, *EBI3*^−/−^, *p28*^−/−^, and WT mice were deeply anaesthetised with sodium pentobarbital (50 mg/kg, i.p.) and then sacrificed with transcardial perfusion with 20 ml of 4% paraformaldehyde in 0.1 M sodium phosphate buffer (PB; pH 7.4). The L4 segment of the SC and the DRG were removed, post-fixed in the same fixative at 4 °C overnight, and placed in 30% sucrose solution for 24 h at 4 °C. Transverse L4 SC sections (30 µm) were cut, rinsed in PBS (3 × 10 min), and preincubated for 30 min with 1% normal donkey serum containing 1% bovine serum albumin and 0.1% Triton X-100 in PBS. Sections were then incubated overnight at 4 °C with the primary antibody. Rabbit anti-Iba1 (1:250, Wako) was used. After rinsing in PBS (3 × 10 min), these sections were incubated with the secondary antibody. Donkey anti-rabbit IgG conjugated Alexa Fluor 488 (1:400, Abcam, Cambridge, UK) was used. TO-PRO-3 (1:200, Molecular Probes, Eugene, OR) was also used for nuclear staining together with the secondary antibody. We randomly selected 3–5 sections of the L4 SC of each mouse for analysis with a LSM 5 Exciter confocal microscope [equipped with 488 nm Argon, 543 nm Helium Neon (HeNe), and 633 nm HeNe lasers] with Zen 2009 software (Carl Zeiss, Oberkochen, Germany). Iba1-positive and TO-PRO-3 positive cells were quantified throughout the dorsal horn.

### Electrophysiology

#### Skin-nerve preparations *in vitro*

*Ex vivo* hind limb skin-saphenous nerve preparations were used for the electrophysiological analysis of nociceptors^65,66^. A single nerve was identified with manual probing of the cutaneous receptive field (RF) with a blunt glass rod. A C-fibre nociceptor with a conduction velocity of <2 m/s was employed for analysis. Background discharge rate was calculated during the control period (60 s) immediately before the mechanical stimulus. The distribution (size and location) of the RFs was mapped on a standardised chart. The size of each RF was measured by calculating the number of pixels in the RF on a chart drawn with Image J software (free software developed by the National Institutes of Health, Bethesda, MD).

Cutaneous C-fibre nociceptors were classified according to their responsiveness to cold and heat stimulation: 1) C-mechanical nociceptors had a high mechanical response threshold with no response to either cold or heat; 2) C-mechano-cold units responded to mechanical and cold stimuli; 3) C-mechano-heat units responded to mechanical and heat stimuli; and 4) C-mechano-cold-heat units were sensitive to mechanical, cold, and heat stimuli.

#### Mechanical stimulation

To analyse the mechanical sensitivity of C-fibre nociceptors, we used a mechanical stimulator (PS-1, manufactured by Aizawa S., Goto College of Medical Arts and Science, Tokyo, Japan) with feedback regulation of force as in our previous study^67^. A ramp-shaped mechanical stimulus, linearly increasing from 0 to 392 mN in 40 s, was applied to the most sensitive point of the identified RF.

#### Application of recombinant IL-27

The application of rIL-27 to *EBI3*^−/−^ mice reduced pain-related behaviours. Therefore, we examined whether externally applied rIL-27 could normalise the facilitated responsiveness of C-fibre nociceptors. We injected rIL-27 (16 µg/kg) intraperitoneally into the *EBI3*^−/−^ mice 1 h before the surgical operation to remove the skin-nerve preparations.

#### Statistical analyses for electrophysiology

Results are expressed as medians with interquartile range (IQR). Summarised mechanical response patterns are expressed as mean ± SEM. Comparisons between WT mice and mice lacking IL-27/WSX-1 signalling were performed with the Kruskal-Wallis test followed by Dunn’s multiple comparison test. Comparisons between *EBI3*^−/−^ mice and *EBI3*^−/−^ +rIL-27 were performed with the Mann-Whitney U test. *P* < 0.05 was considered significant.

### Data Availability

The datasets generated during and/or analysed during the current study are available with the corresponding author, and can be accessed on reasonable request.

## Electronic supplementary material

Supplementary Information
