# Supplementary material for: Interleukin-27 controls basal pain threshold in physiological and pathological conditions
Source: Sci Rep. 2018 Jul 23;8:11022. doi: 10.1038/s41598-018-29398-3 (PMC6056516; doi:10.1038/s41598-018-29398-3)
Supplement: Supplementary file 1 — Supplementary Information [file 41598_2018_29398_MOESM1_ESM.pdf]

Supplementary Information

Interleukin-27 controls basal pain threshold in physiological and pathological conditions

Tomoko Sasaguri[1], Toru Taguchi[2, 3], Yuzo Murata[4], Kimiko Kobayashi[5], Sayaka Iizasa[6], Ei'ichi Iizasa[7], Makoto Tsuda[8], Naomi Hirakawa[1], Hiromitsu Hara[7], Hiroki Yoshida[9]*, Toshiharu Yasaka[7]*

[1]*Department of Anesthesiology & Critical Care Medicine, Faculty of Medicine, Saga University, 5-1-1 Nabeshima, Saga 849-8501, Japan*

[2]*Department of Physical Therapy, Niigata University of Health and Welfare, 1398 Shimami-cho, Kita-ku, Niigata 950-3198, Japan.*

[3]*Department of Neuroscience II, Research Institute of Environmental Medicine, Nagoya University, Furo-cho, Chikusa-ku, Nagoya 464-8601, Japan.*

[4]*Division of Histology and Neuroanatomy, Department of Anatomy & Physiology, Faculty of Medicine, Saga University, 5-1-1 Nabeshima, Saga 849-8501, Japan*

[5]*Department of Anatomy and Neuroscience, Hyogo College of Medicine, 1-1 Mukogawa-cho, Nishinomiya, Hyogo 663-8501, Japan*

[6]*Department of Biological Science and Technology, The United Graduate School of Agricultural Sciences, Kagoshima University, 1-21-24 Korimoto, Kagoshima 890-8580, Japan*

[7]*Department of Immunology, Graduate School of Medical and Dental Sciences, Kagoshima University, 8-35-1 Sakuragaoka, Kagoshima 890-8544, Japan*

[8]*Department of Molecular and System Pharmacology, Graduate School of Pharmaceutical Science, Kyushu University, 3-1-1 Maidashi, Higashi-ku, Fukuoka 812-8582, Japan*

[9]*Division of Molecular and Cellular Immunoscience, Department of Biomolecular Sciences, Faculty of Medicine, Saga University, 5-1-1 Nabeshima, Saga 849-8501, Japan*

*Correspondence should be addressed to T.Y. (email: yasaka@m.kufm.kagoshima-u.ac.jp) and H.Y. (email: yoshida@med.saga-u.ac.jp)

Supplementary information includes:

Supplementary Methods, Supplementary References, Supplementary Figures S1-S10, and Supplementary Table S1

Supplementary Methods

Assessment of motor function and anxiety-like behaviour. To exclude the possible influence of abnormalities in motor function and/or anxiety that might affect pain-related behaviour, mice lacking IL-27/WSX-1 signalling were evaluated with the open field test and the rotarod test. We placed 8-week-old mice (10 animals/group) individually into the centre of the field (50 cm in diameter; Muromachi kikai, Tokyo, Japan) which was brightly illuminated by overhead fluorescent lighting (500 lx). The mice were not given time to habituate and were observed for 5 min via a video camera with a computational tracking system (ANY-maze; Stoelting Co., Wood Dale, IL). We analysed locomotor activity and anxiety-like behaviour, expressed as the total distance travelled and time spent in the centre area, respectively[1,2]. We also used a rotarod (MK-630, Muromachi kikai) to assess motor coordination and balance. We conducted the rotarod test according to the manufacturer's instructions. One hour before data collection, mice were placed on a static cylinder for 5 min for habituation. Subsequently, they were placed on a rotating cylinder (5 rpm) for 5 min. During the test, mice were placed on a constant-speed rotarod (12 rpm) for a maximum of 300 s, and latency to falling off the rotarod was measured.

Immunohistochemistry. The same procedure described in the main text was used to prepare sections from naïve WT, *WSX-1*$^{-/-}$, *EBI3*$^{-/-}$, and *p28*$^{-/-}$ mice. Transverse L4 spinal cord (SC) sections (30 μm) and dorsal root ganglion (DRG) sections (20 μm) were cut and rinsed in PBS (3 × 10 min). Next, they were preincubated with 1% normal donkey serum containing 1% bovine serum albumin and 0.1% Triton X-100 in PBS for 30 min. Subsequently, sections were incubated overnight at 4°C with primary antibodies: rabbit anti-calcitonin gene-related peptide (CGRP, 1:4,000, Cambridge Research Biochemicals), mouse anti-neurofilament 200 (NF200, 1:2,000, Sigma-Aldrich, St. Louis, MO), isolectin B4 (IB4, biotin conjugate; 1:2,000, Sigma-Aldrich), rabbit anti-ionised calcium-binding adapter molecule-1 (Iba1, 1:250, Wako Pure Chemical Industries, Osaka, Japan), goat anti-[SRY (sex determining region Y)-box 9] (SOX9, 1:500, R&D Systems, Inc., Minneapolis, MN), and chicken anti-glial fibrillary acidic protein (GFAP, 1:500, EMD Millipore Corporation, Temecula, CA). After rinsing in PBS (3 × 10 min), the sections were incubated with the secondary antibodies: donkey anti-rabbit IgG conjugated Alexa Fluor 488 (1:400, Abcam, Cambridge, UK), and/or donkey anti-mouse IgG conjugated Alexa Fluor 546 (1:400, Thermo Fisher Scientific K.K., Osaka, Japan), or donkey anti-goat IgG conjugated

Alexa Fluor 546 (1:400, Molecular Probes, Eugene, OR) alone or in some cases with donkey anti-chicken IgY conjugated Alexa Fluor 647 (Jackson ImmunoReserach Laboratories, Inc., West Grove, PA). TO-PRO-3 (1:200, Molecular Probes) was also used for nuclear staining together with the secondary antibodies for spinal cord sections. The same procedure described in the main text was used to analyse these sections. SOX9, but not GFAP was used for the marker of astrocytes for cell counting, because we found it more suitable to identify individual astrocytes (Supplementary Fig. 4c).

Real-time PCR analysis. Mice were deeply anaesthetised with pentobarbital (50 mg/kg) and perfused transcardially with 20 ml of RNAlater (Invitrogen, San Diego, CA). Total RNA was prepared with RNeasy Lipid Tissue Mini kit (Qiagen, Tokyo, Japan) according to the manufacturer's instructions. It was then treated with RT Grade DNase (Nippon Gene, Tokyo, Japan), and used for reverse transcription with a ReverTra Ace qPCR RT Kit (TOYOBO CO., LTD. Osaka, Japan) to produce cDNA. Quantitative PCR was performed with a PicoReal 96 Real-Time PCR System (Thermo Fisher Scientific K.K.) by using SYBR green I dye detection (TOYOBO). The cycling conditions were as follows: 95°C for 1 min, followed by 40 cycles of 95°C for 5 s, and 60°C for 30 s. Expression levels of each gene were normalised to the values for the mouse glyceraldehyde-3-phosphate dehydrogenase (GAPDH) gene. The sequences of primers are shown in Supplementary Table S1.

Drug application. To assess whether the hypersensitivity observed in mice lacking IL-27/WSX-1 signalling was mediated by microglia, we applied an intraperitoneal (i.p.) injection of 50 mg/kg minocycline, a microglial inhibitor, or saline, to *WSX-1*$^{-/-}$, *EBI3*$^{-/-}$, *p28*$^{-/-}$, and WT mice. We determined the dose of minocycline employed in this study by reference to a previous study[3]. As a positive control experiment, the same dose of minocycline was also delivered by i.p. injection to neuropathic WT mice (7 days after spinal nerve injury). The von Frey test was used to evaluate mechanical sensitivity, and data were collected before and 4 hr after minocycline injection.

To test whether the chronic pain-like hypersensitivities in IL-27/WSX-1 signalling-deficient mice can be treated with commonly used drugs for pain relief, these mice were received systemic injection of non-steroidal anti-inflammatory drug (NSAIDs) or opioid agonists. One of NSAIDs, Ibuprofen (120 mg/kg), or saline was injected (i.p.) to *EBI3*$^{-/-}$ mice. The hot-plate test (at 48ºC) was used to evaluate the

effect of ibuprofen, and data were collected before and after the injection. To assess the effects of opioid agonists, *WSX-1*$^{-/-}$ and *p28*$^{-/-}$ mice received a subcutaneous injection of [D-Ala², N-MePhe⁴, Gly-ol⁵]enkephalin (DAMGO, a selective μ-opioid receptor agonist) (6 mg/kg), [D-Pen², D-Pen⁵]enkephalin (DPDPE, a selective δ-opioid receptor agonist) (6 mg/kg), (5α,7α,8β)- (+)-N-Methyl-N-[7-(1-pyrrolidinyl)-1-oxaspiro[4.5]dec-8-yl]benzeneacetamide (U-69593, a κ-opioid receptor agonist) (120 μg/kg), or vehicle (saline or 1% dimethyl sulfoxide [DMSO] in saline). We determined the doses of opioid receptor agonists by reference to a previous study[4]. The effects of these drugs were evaluated with the hot-plate test (at 48°C), and data were obtained before and after the injection.

To test whether the effects of recombinant IL-27 (rIL-27) are mediated by the opioid system, we used naloxone, a non-selective antagonist of opioid receptors. First, *p28*$^{-/-}$ mice received an i.p. injection of rIL-27 (16 μg/kg). Then, 75 min later they received another i.p. injection of naloxone (1.2 mg/kg) or saline. We determined the dose of naloxone by reference to a previous study[4]. We used the hot-plate test (at 48°C).

To test whether hypersensitive phenotypes observed in mice lacking IL-27/WSX-1 signalling were mediated by the adenosine triphosphate (ATP) system, non-selective P2 receptor antagonist, pyridoxal-phosphate-6-azo (benzene-2,4-disulfonic acid) (PPADS) tetrasodium salt (30 mg/kg), or saline by injecting (i.p.) *WSX-1*$^{-/-}$ and *p28*$^{-/-}$ mice. We determined the dose of PPADS by reference to a previous study[5]. The von Frey test was used to evaluate effects of PPADS, and data were obtained before, and 1, 3, and 6 hr after the injection.

Drugs and chemicals. The following drugs and chemicals were used. Ibuprofen was purchased from Wako. DAMGO, U-69593, naloxone hydrochloride dehydrate, PPADS, and minocycline were obtained from Sigma-Aldrich. DPDPE was obtained from Abcam. All drugs except U-69593 were dissolved or diluted in saline. U-69593 was first dissolved in DMSO and then diluted in saline (1:100).

***In situ* hybridisation histochemistry (ISHH).** Mice were sacrificed by decapitation under deep ether anaesthesia. The tissues were removed, rapidly frozen with powdered dry ice, and cut on a cryostat to a thickness of 8–14 μm. By using the enzyme-digested clones, we synthesised ^{35}S UTP-labelled mouse *WSX-1* antisense and sense cRNA probes (cDNA nucleotides 19–643, accession number NM_016671.3;

in pCRII TOPO vector). The protocol for ISHH has been described in detail previously[6].

Statistical analyses. Statistical analyses of results were performed with the Mann-Whitney U test, Kruskal-Wallis test with Steel-Dwass post hoc test, two-way ANOVA followed by Tukey's post hoc tests, or Kruskal-Wallis test followed by Dunn's multiple comparison test. A P value less than 0.05 was considered statistically significant.

Supplementary References

1. Tamada, K. *et al.* Decreased exploratory activity in a mouse model of 15q duplication syndrome; implications for disturbance of serotonin signaling. *PLoS One* **5**, e15126 (2010).
2. Archer, J. Tests for emotionality in rats and mice: a review. *Anim. Behav.* **21**, 205-235 (1973).
3. Bastos, L.F. *et al.* A minocycline derivative reduces nerve injury-induced allodynia, LPS-induced prostaglandin E2 microglial production and signaling via toll-like receptors 2 and 4. *Neurosci. Lett.* **543**, 157-162 (2013).
4. Towett, P.K., Kanui, T.I., Maloiy, G.M., Juma, F. & Olongida Ole Miaron, J. Activation of micro, delta or kappa opioid receptors by DAMGO, DPDPE, U-50488 or U-69593 respectively causes antinociception in the formalin test in the naked mole-rat (Heterocephalus glaber). *Pharmacol., Biochem.,Behav.* **91**, 566-572 (2009).
5. Magni, G., Merli, D., Verderio, C., Abbracchio, M.P. & Ceruti, S. P2Y2 receptor antagonists as anti-allodynic agents in acute and sub-chronic trigeminal sensitization: role of satellite glial cells. *Glia* **63**, 1256-1269 (2015).
6. Kobayashi, K. *et al.* P2Y12 receptor upregulation in activated microglia is a gateway of p38 signaling and neuropathic pain. *J. Neurosci.* **28**, 2892-2902 (2008).

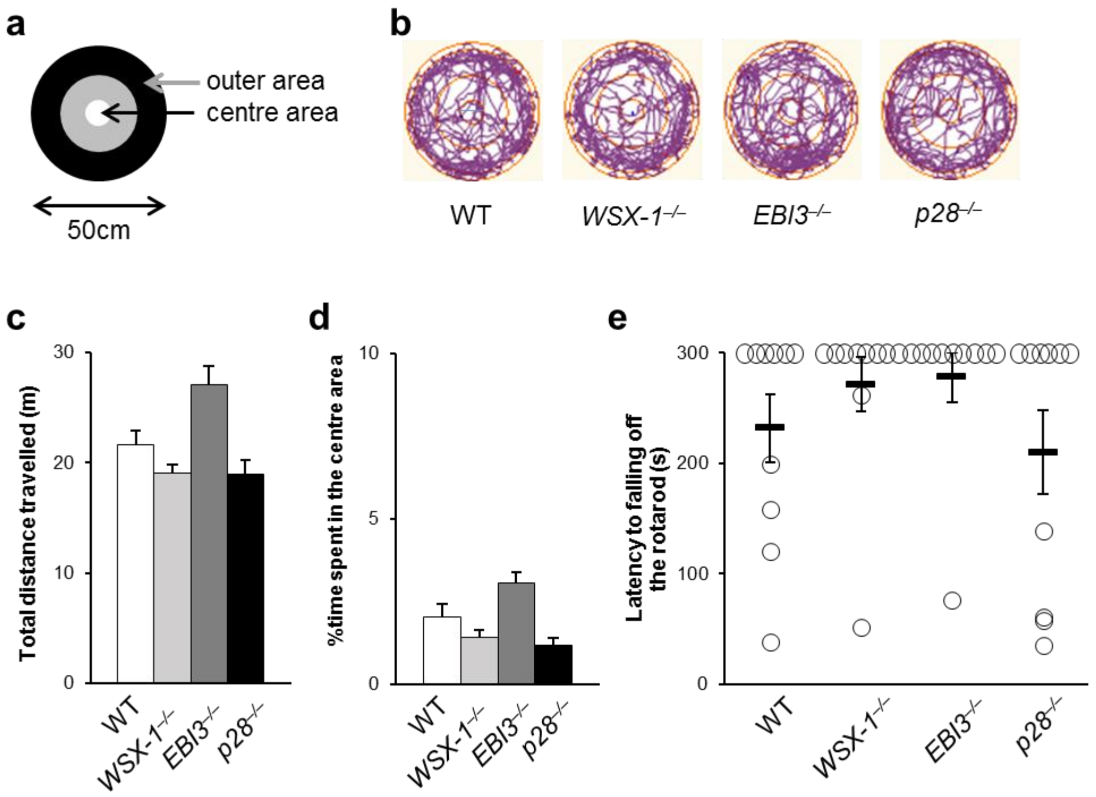

Supplementary Figure S1. Mice lacking IL-27/WSX-1 signalling show normal locomotor activity and anxiety-like behaviour. (**a**) Schema of the field used. (**b**, **c**) An open-field test for a total of 5 min showed no difference in path length or (**d**) percentage of time spent in the centre area. The images in **b** are representative of ten experiments. (**e**) Latency to fall off the rotarod, obtained from WT, *WSX-1*$^{-/-}$, *EBI3*$^{-/-}$, and *p28*$^{-/-}$ mice. *n* = 10 animals/group. All data are expressed as mean ± SEM.

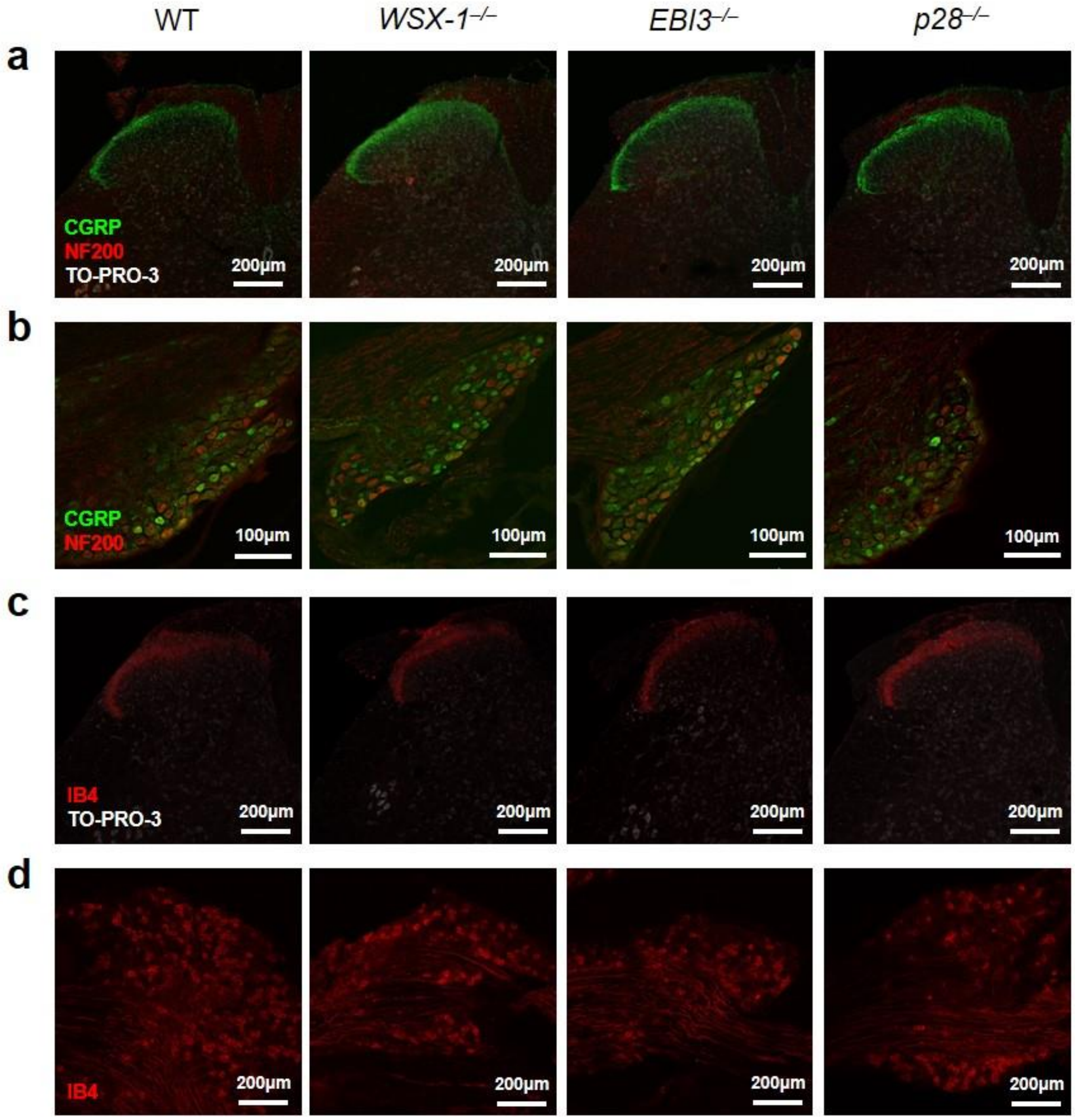

Supplementary Figure S2. Normal distribution of primary afferents in the spinal dorsal horn and the DRG of mice lacking IL-27/WSX-1 signalling. (**a**, **b**) CGRP and NF200 immunoreactivity and (**c**, **d**) IB4 binding in a transverse section of (**a**, **c**) the dorsal horn and (**b**, **d**) the DRG from the L4 segment of naïve WT, *WSX-1*$^{-/-}$, *EBI3*$^{-/-}$, and *p28*$^{-/-}$ mice. The TO-PRO-3 was used for nuclear staining. Images are representative of five experiments.

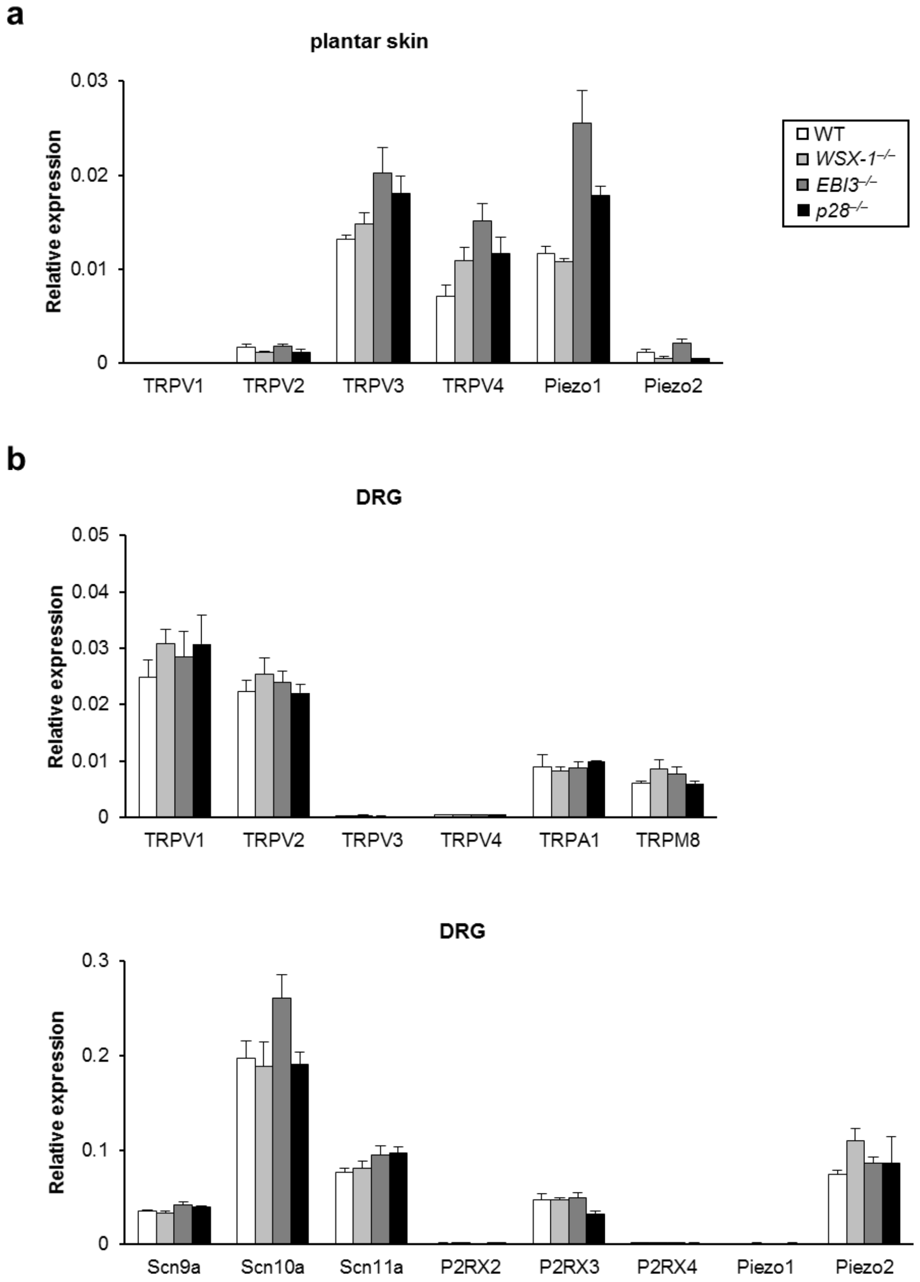

Supplementary Figure S3. Preserved expression level of major ion channels in the plantar skin and DRG of IL-27/WSX-1 signalling deficient mice. Relative expression of mRNA encoding various ion channels including TRP, voltage-gated sodium, P2X and Piezo channels in (**a**) plantar skin and/or (**b**) DRG of WT, *WSX-1*$^{-/-}$, *EBI3*$^{-/-}$, and *p28*$^{-/-}$ mice. n = 3 animals/group. All data are expressed as mean ± SEM. Values of the Relative expression of TRPV1 in the plantar skin are less than 0.00001, and those of TRPV3, TRPV4, P2XR2, P2XR4, and Piezo1 in the DRG are less than 0.002.

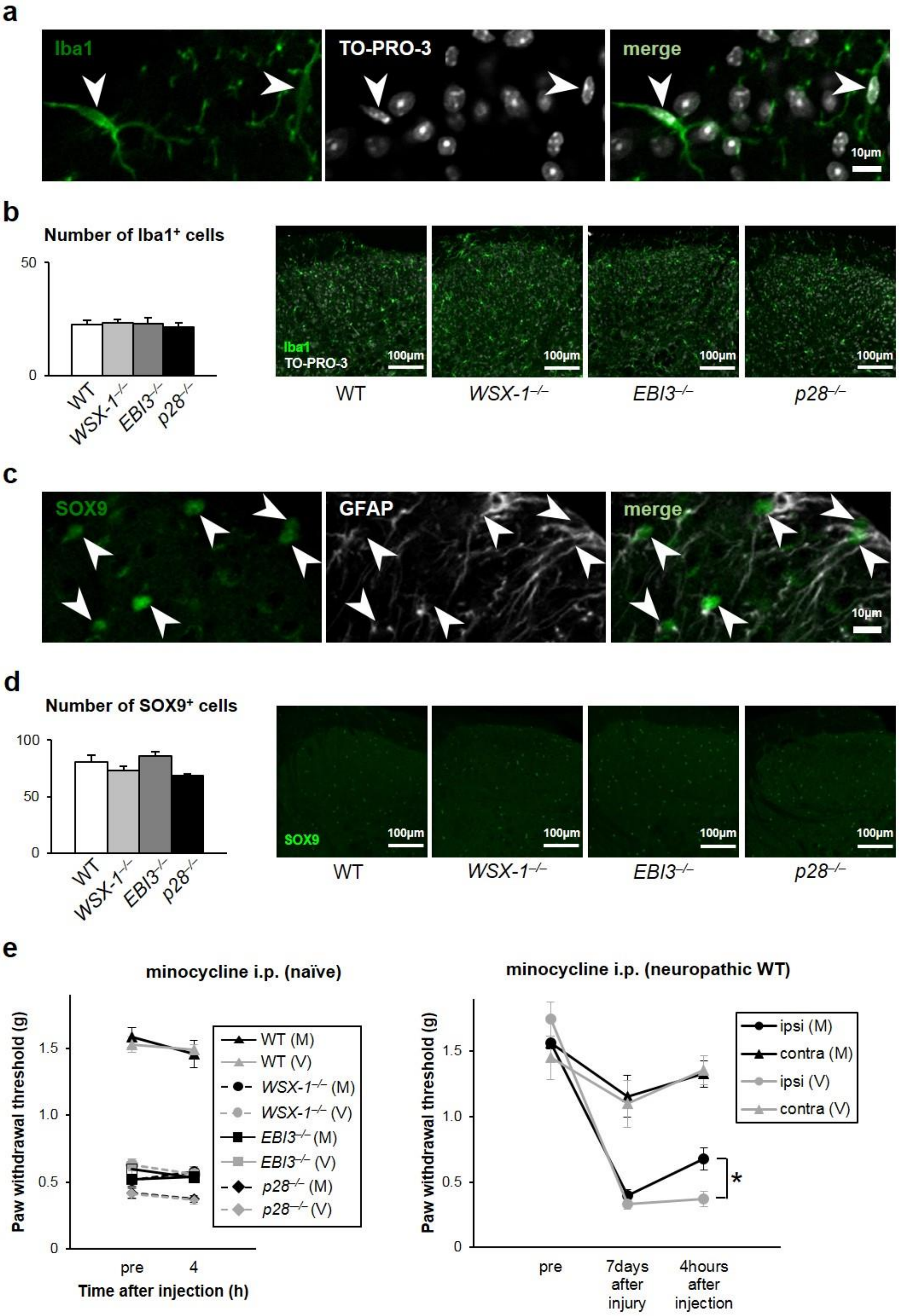
a
Iba1
TO-PRO-3
merge
10μm
b
Number of Iba1+ cells
50
0
WT
WSX-1−/−
EBI3−/−
p28−/−
Iba1
TO-PRO-3
100μm
c
SOX9
GFAP
merge
10μm
d
Number of SOX9+ cells
100
50
0
SOX9
100μm
e
minocycline i.p. (naïve)
Paw withdrawal threshold (g)
1.5
1.0
0.5
0
pre
4
Time after injection (h)
WT (M)
WT (V)
WSX-1−/− (M)
WSX-1−/− (V)
EBI3−/− (M)
EBI3−/− (V)
p28−/− (M)
p28−/− (V)
minocycline i.p. (neuropathic WT)
ipsi (M)
contra (M)
ipsi (V)
contra (V)
*
7days after injury
4hours after injection

Supplementary Figure S4. Normal appearance of glial cells in the spinal dorsal horn of mice lacking IL-27/WSX-1 signalling. (**a**) The method used to count microglia. Cells were counted only when Iba1-immunoreactive process was colocalised with nuclear staining (TO-PRO-3, white). Arrowheads indicate cells counted. (**b**) Number of Iba1-immunoreactive cells in a transverse section through the dorsal horn from the L4 segment of naïve WT, *WSX-1*$^{-/-}$, *EBI3*$^{-/-}$, and *p28*$^{-/-}$ mice and corresponding images showing Iba1-immunoreactivity (green) with nuclear staining (white) are shown. The images are representative of five experiments. (**c**) Counting astrocytes using SOX9-staining. SOX9-immunoreactivities are located in astrocytic nuclei whereas GFAP-immunoreactivities are located in their processes but not cell bodies. Note that all of the SOX9-immunoreactive nuclei (arrowheads) are associated with GFAP-immunoreactive processes, and some of these are difficult to identify by GFAP-staining alone. (**d**) Number of SOX9-immunoreactive cells in a transverse section thorough the dorsal horn from the L4 segment of each strain and corresponding images showing SOX9 immunoreactivities (green) are shown. The images are representative of five experiments. (**e**) Paw withdrawal threshold measured with the von Frey test before and after i.p. injection of minocycline (50 mg/kg, (M)) or saline (V) into naïve WT, *WSX-1*$^{-/-}$, *EBI3*$^{-/-}$, and *p28*$^{-/-}$ mice (left) and neuropathic WT mice that had received left L4 spinal nerve injury 7 days before (right). n = 3–5 animals/group. *P< 0.05 vs. saline control. Two-way ANOVA followed by Tukey's post hoc tests (**e**). All data are expressed as mean ± SEM.

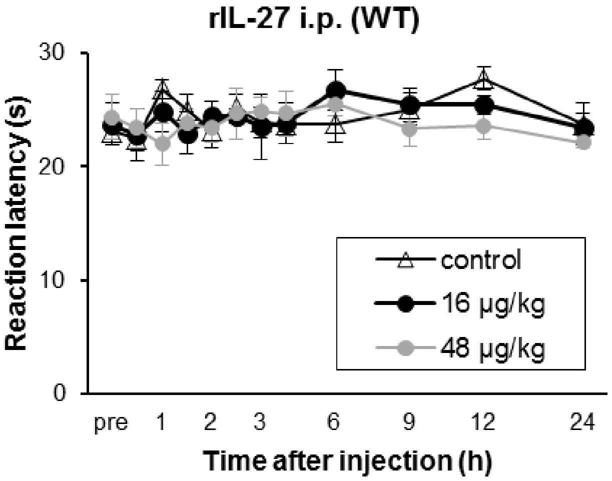

Supplementary Figure S5. Application of rIL-27 does not affect thermal sensation in WT mice. Reaction latency measured with the hot-plate test before and after i.p. injection of saline, 16 μg/kg rIL-27, or 48 μg/kg rIL-27 into WT mice. *n* = 5 animals/group. All data are expressed as mean ± SEM.

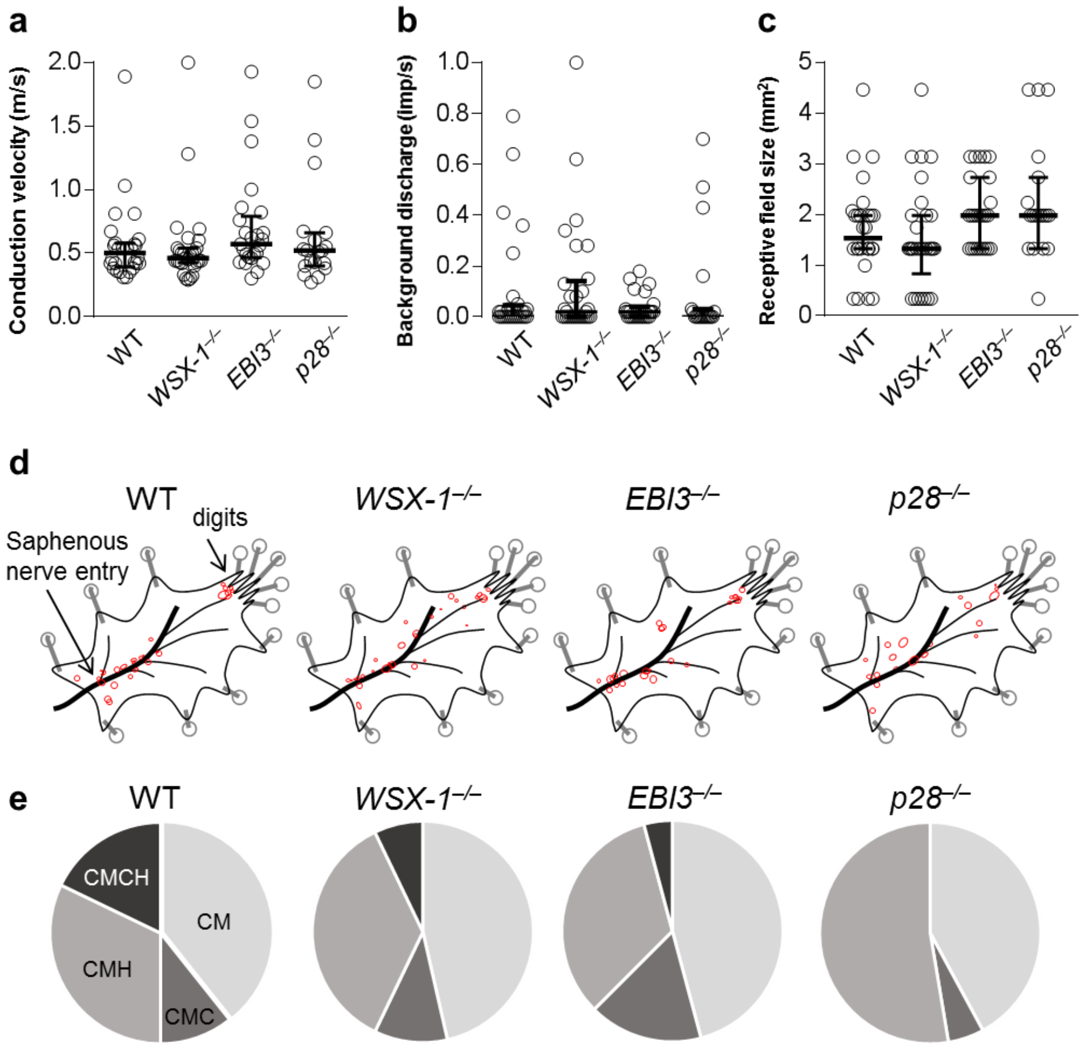

Supplementary Figure S6. Preserved general characteristics of C-fibre nociceptors recorded with an *ex vivo* skin-nerve preparation in mice lacking IL-27/WSX-1 signalling. (**a**) Conduction velocity, (**b**) background discharge rate, and (**c**) the size of receptive field in WT, *WSX-1*$^{-/-}$, *EBI3*$^{-/-}$, and *p28*$^{-/-}$ mice. (**d**) Representative distribution of the receptive fields. (**e**) Proportion of receptor types in C-nociceptors. CM, C-mechanical nociceptors; CMC, C-mechano-cold units; CMH, C-mechano-heat units; and CMCH, C-mechano-cold-heat units. No significant differences were observed in the general characteristics among animal groups. WT (n = 28), *WSX-1*$^{-/-}$ (n = 29), *EBI3*$^{-/-}$ (n = 25), and *p28*$^{-/-}$ (n = 19). (**a**)–(**c**) All data are expressed as median with interquartile range (IQR).

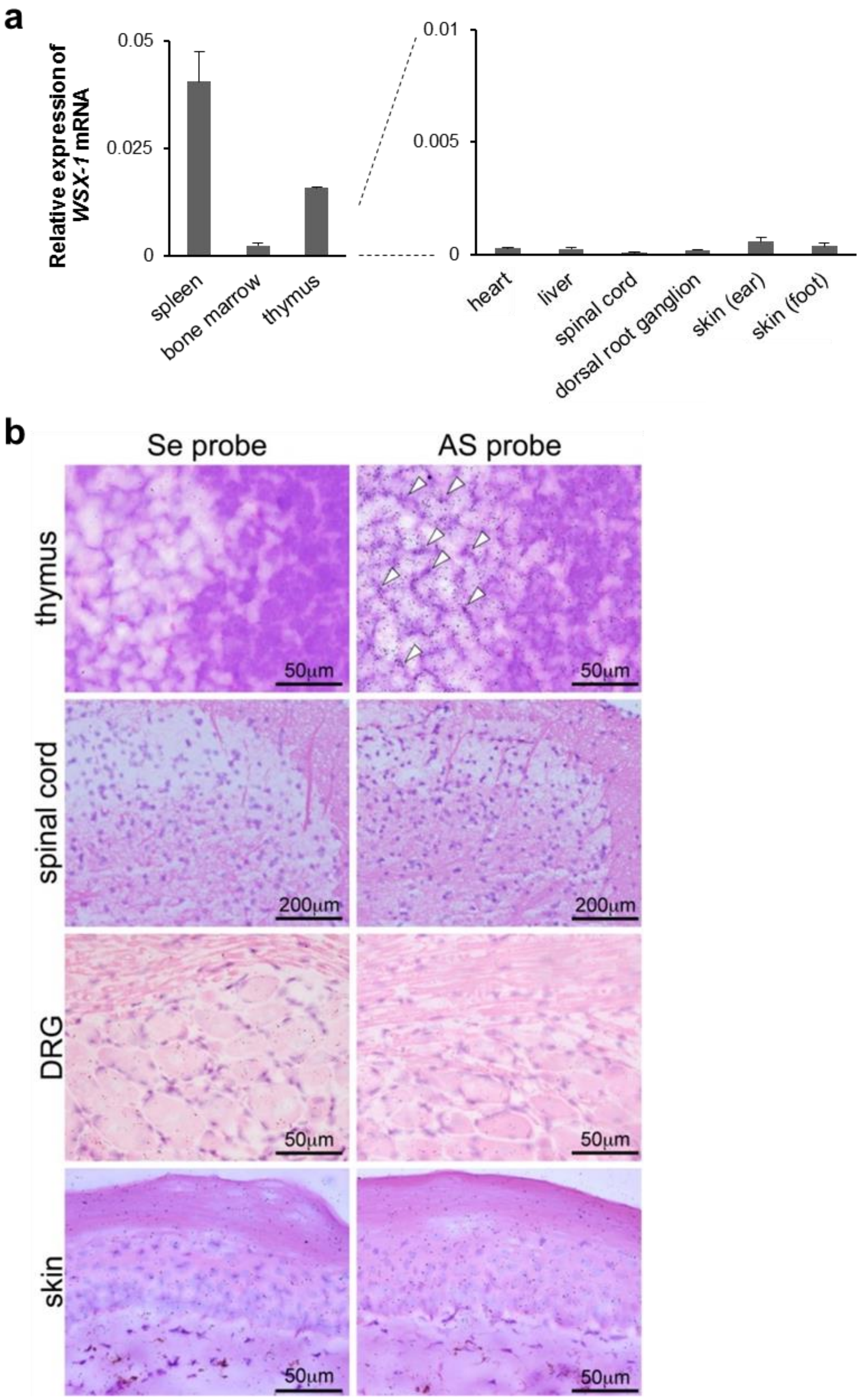
a
Relative expression of WSX-1 mRNA
0.05
0.025
0
spleen
bone marrow
thymus
0.01
0.005
0
heart
liver
spinal cord
dorsal root ganglion
skin (ear)
skin (foot)
b
Se probe
AS probe
thymus
spinal cord
DRG
skin
50μm
200μm

Supplementary Figure S7. Limited expression of *WSX-1* mRNA in the sensory pathway. (**a**) Relative expression of *WSX-1* mRNA in various tissues of WT mice. *n* = 3 animals/group. All data are expressed as means ± SEM. (**b**) *In situ* hybridisation histochemistry of *WSX-1* mRNA. Photomicrographs show hybridisations in the thymus, spinal cord, DRG, and footpad skin with sense probe (left panel) and anti-sense probe (right panel). *WSX-1* mRNA signals highly expressed in the thymic medulla (arrowheads) after a week of autoradiographic exposure, whereas only background levels of grains were observed on the spinal cord, DRG, and footpad skin after 9 weeks exposure. The sections were counterstained with haematoxylin and eosin.

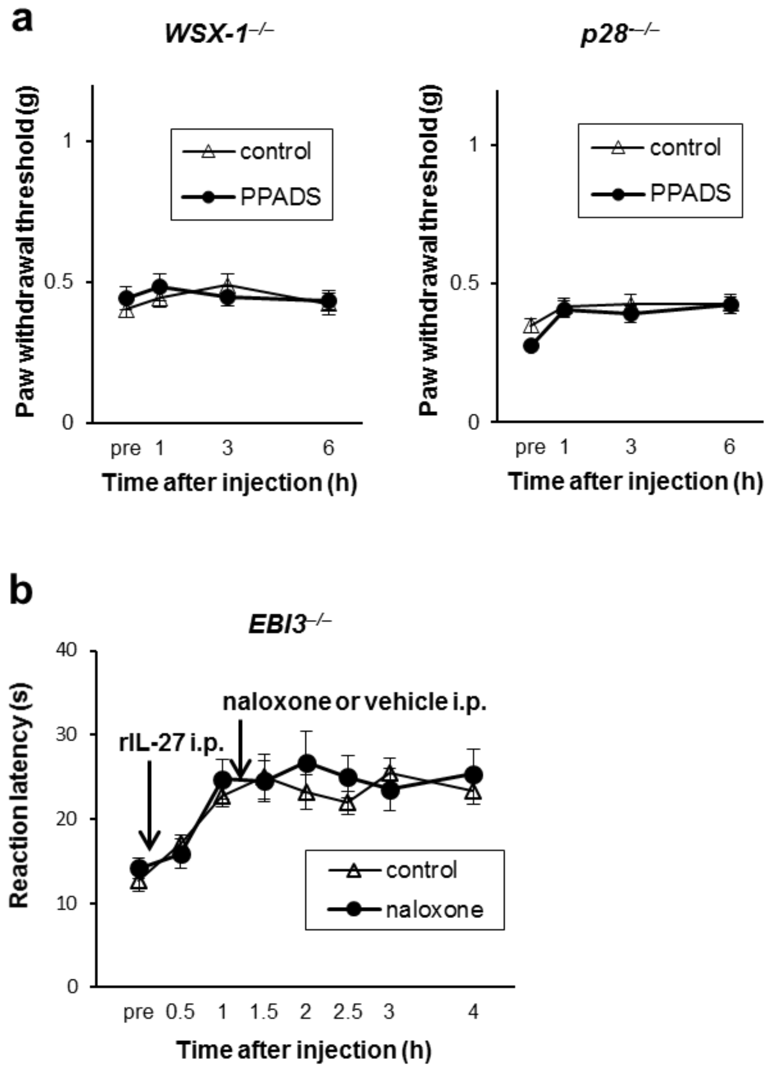

Supplementary Figure S8. Hypersensitive phenotypes in mice lacking IL-27/WSX-1 signalling are likely independent of the opioid or ATP system. (**a**) Fifty percent paw withdrawal threshold obtained by the von Frey test in *WSX-1*$^{-/-}$ and *p28*$^{-/-}$ mice before and after i.p. injection of PPADS or saline. (**b**) Reaction latency obtained from a hot-plate test before and after i.p. injection of rIL-27 into *EBI3*$^{-/-}$ mice that received another i.p. injection of naloxone or saline 75 min after rIL-27 injection. n = 5 animals/group. All data are expressed as mean ± SEM.

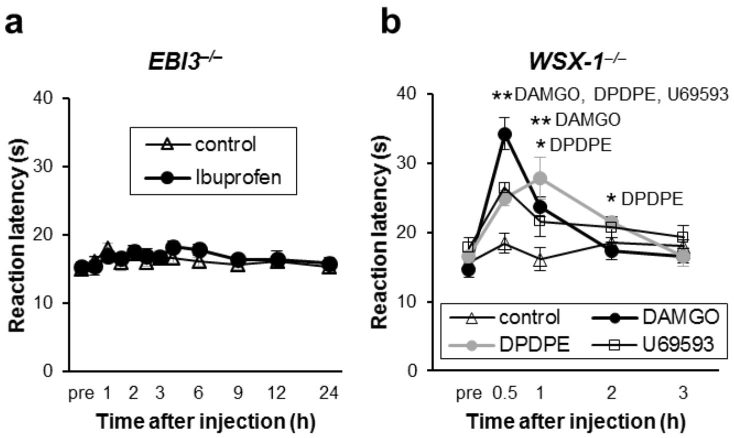

Supplementary Figure S9. Opioid agonists but not NSAIDs provide effective treatment for pain relief to chronic pain-like basal hypersensitivity in mice lacking IL-27/WSX-1 signalling. (**a**) Reaction latency obtained from the hot-plate test before and after a single i.p. injection of ibuprofen (120 mg/kg) or saline into *EBI3*$^{-/-}$ mice. (**b**) Reaction latency obtained from the hot-plate test before and after single subcutaneous injection of saline or three kinds of opioid receptor agonist (DAMGO, DPDPE, and U69593) into *WSX-1*$^{-/-}$ and *p28*$^{-/-}$ mice. $n = 5$ animals/group. *$P < 0.05$, **$P < 0.01$ vs. saline control. Two-way ANOVA followed by Tukey's post hoc tests. All data are expressed as mean ± SEM.

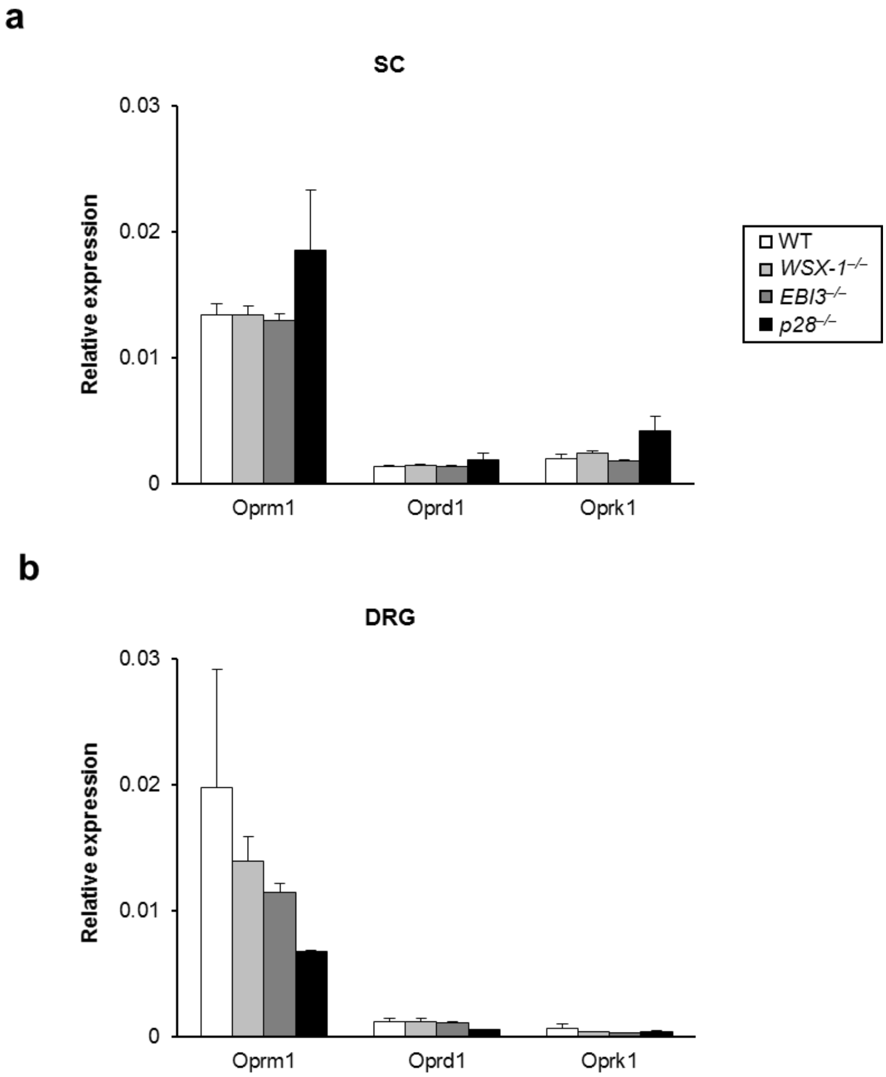

Supplementary Figure S10. Preserved expression of opioid receptors in the spinal cord and DRG of IL-27/WSX-1 signalling deficient mice. Relative expression of mRNA encoding μ-,δ-, or κ-opioid receptors in (**a**) spinal cord (SC) and (**b**) DRG of WT, *WSX-1*$^{-/-}$, *EBI3*$^{-/-}$, and *p28*$^{-/-}$ mice. *n* = 3 animals/group. All data are expressed as mean ± SEM.

Supplementary Table S1. Primer sets used for real-time PCR analysis.

Gene	Accession number	Forward primer sequence<br>Reverse primer sequence
TRPV1	NM_001001445	TGTGGAGGTGGCAGAT<br>CTTCAGTGTGGGGTGG
TRPV2	NM_011706	CTGCTGAAAGTTGGCACCAAAG<br>CTCCCATGCAGCCCAGTTTAC
TRPV3	NM_145099	CCAAGATGGGCAAGGCTGA<br>TCGTTGTGTCTACATTGGTGAGGTC
TRPV4	NM_022017	ACTGTGGGCAAGAGCTCAGATG<br>CTCTTGCCAGGGTCCTCGTTA
TRPM8	NM_134252	AGGCATCGGTTTAGACAACTGGAC<br>AGCATCGCCAGCCTTACTTGA
TRPA1	NM_177781	GTCCAGGGCGTTGTCT<br>CGTGATGCAGAGGACAGAGAT
Piezo1	HQ215520	CTTTATCATCAAGTGCAGCCGAGA<br>CCAGATGATGGCGATGAGGA
Piezo2	NM_001039485	ACTCAGCAAAGCAACTACGTGGAC<br>GAGCCATCCGAAGCAGAATGA
Scn9a	NM_001290674	GCAGTGGTAAGCCAGACAATACAGA<br>CTCACCATTAAGTTGCCCAAACA
Scn10a	NM_001205321	GCTTTGTGTTGTTTGTGACCAGAGA<br>ATTGCCTGAGACCACCAGAAATG
Scn11a	NM_011887	TGGGCGAAGACGACTTTGAG<br>GAAACTGAAACCTGTTGGGCTTG
P2RX2	NM_001310701	CCATCTTCAGGCTGGGCTTC<br>TTCAGACAAGTCCAGGTCACAGTTC
P2RX3	NM_145526	TCTCCAGCAGAGACATCAGCA<br>GGGAGCATCTTGGTGAACTCAG
P2RX4	NM_011026	TCCTGGCTTACGTCATTGGG<br>AGAAGTGTTGGTCACAGCCA
Oprm1	NM_001302795	TGGTACTGGGAGAACCTGCT<br>ATGCGGACACTCTTGAGTCG
Oprd1	NM_013622	GTCATGTTTGGCATCGTCCG<br>CAAGTACTTGGCGCTCTGGA
Oprk1	NM_001204371	GCCACCCTGTGAAAGCTTG<br>TTCCCTGACTTTGGTGCCTC
WSX-1	NM_016671	GGACCAGGAAACCGTTGGAGT<br>GTTGAGCTTGTCCAGGCTGTC
GAPDH	GU214026	AACTTTGGCATTGTGGAAGG<br>ACACATTGGGGGTAGGAACA
